# Identification and characterization of RacX, a new broad-specificity amino acid racemase from a novel taxon within the order *Flavobacteriales*

**DOI:** 10.1128/aem.02015-25

**Published:** 2025-12-22

**Authors:** Li Hu, Xin-Yun Tan, Yu-Qi Ye, Xin-Yu Liu, Yu-Zhu Li, Jing-Yao Wang, Ting-Ran Zhang, Zong-Jun Du, Meng-Qi Ye

**Affiliations:** 1 Marine College, Shandong University, Weihai, Shandong, People's Republic of China; 2 Weihai Research Institute of Industrial Technology of Shandong University 154528, Weihai, People's Republic of China; 3 Shenzhen Research Institute, Shandong University, Shenzhen, People's Republic of China; Kyoto University, Kyoto, Japan

**Keywords:** amino acid racemase, broad specificity, catalytic efficiency, d-amino acid, *Halocolaceae *fam. nov, *Halocola ammonii*

## Abstract

Amino acid racemases are pivotal for d-amino acid (DAA) biosynthesis with wide-ranging biotechnological applications, yet their industrial deployment is hindered by narrow substrate specificity and instability. Here, we report the discovery of *Halocola ammonii* gen. nov., sp. nov. DA487^T^, a novel taxon within the proposed family *Halocolacceae* fam. nov. (order *Flavobacteriales*), isolated from hypersaline sediments. Genomic analysis revealed a robust DAA metabolic network, including a putative broad-specificity racemase RacX. Biochemical characterization demonstrated RacX’s exceptional catalytic efficiency (*k*_cat_*/K_m_* = 151.2 s^−1^ mM^−1^ for l-Lys, *k*_cat_*/K_m_* = 17.8 s^−1^ mM^−1^ for d-Lys) and broad substrate spectrum (15/17 tested l-amino acids). Homology modeling and mutagenesis identified Ala79 and Cys193 as putative catalytic residues, based on structural conservation with EcL-DER. Remarkably, the A79C variant enhanced the reverse reaction efficiency (d-Lys → l-Lys) by 44%, effectively shifting the enzyme’s catalytic bias and the resulting steady-state ratio of enzyme-bound species. Computational docking suggested that Asn80, Thr81, Asn121, and Thr124 may modulate substrate binding, though experimental structural validation is required. The thermostability-lability tradeoff ($T_{\text{1/2}}^{\text{55°C}}\;=\;\text{ 70 min}$) highlights targets for protein engineering. Our findings not only expand the phylogenetic diversity of microbial racemases but also identify a promising biocatalyst candidate for industrial DAA production.

**IMPORTANCE:**

Microbial adaptations to extreme environments serve as a valuable source of novel biocatalysts with potential for sustainable industrial applications. In this study, we characterized *Halocola ammonii* DA487ᵀ, a halophilic bacterium representing the novel family *Halocolaceae* within the order *Flavobacteriales*, and identified a broad-specificity amino acid racemase, RacX. RacX demonstrates exceptional catalytic efficiency (*k*_cat_*/K_m_* up to 151.2 s⁻¹ mM⁻¹ for l-Lys) across multiple amino acids and exhibits remarkable stability under neutral and alkaline conditions (pH 7.0–9.0)—properties intrinsically linked to its high-salt ecological niche. Unlike most known racemases from neutrophilic organisms, RacX originates from an understudied phylogenetic lineage and displays unique mechanistic features, including a strong innate bias toward d-amino acid (DAA) production that can be rationally reprogrammed via single-residue substitution (e.g., A79C). These functional and evolutionary insights, combined with its halotolerance and broad substrate scope, position RacX as a promising and engineerable biocatalyst for industrial processes requiring operation under high-salt or alkaline conditions, such as the synthesis of DAA precursors for antibiotics.

## INTRODUCTION

The reciprocal conversion between l-amino acids and d-amino acids (DAAs) is mostly carried out by racemases. These enzymes change the chirality of the α-carbon atom in amino acids by means of a deprotonation/reprotonation pathway. Generally, amino acid racemases fall into two groups: enzymes independent of pyridoxal 5′-phosphate (PLP) and enzymes reliant on PLP. For example, alanine racemase (1) and arginine racemase (2) require PLP, which forms a Schiff base in combination with the amino acid substrate. Conversely, PLP-independent enzymes encompass aspartate racemase and glutamate racemase which are sourced from *Escherichia coli* or *Lactobacillus* (3, 4). Despite the extensive study of amino acid racemases from a variety of bacteria, the discovery of new ones remains possible. As an illustration, RacX, originating from *Bacillus subtilis,* and YgeA, from *Escherichia coli* MG1655, have been identified as novel amino acid racemases characterized by broad substrate specificity (5). Further elucidating YgeA’s (also known as EcL-DER) physiological function, recent studies confirm that this PLP-independent racemase acts as a homoserine racemase, converting l-Hse to d-Hse *in vivo* and preventing d-Hse accumulation. Its deficiency impairs bacterial growth (rescued by l-Met/l-Thr/l-Ile) and biofilm formation, revealing YgeA’s dual role in amino acid biosynthesis and biofilm modulation for environmental adaptation (6). Furthermore, based on genome identification and enzyme characterization, it was shown that MalY, sourced from *Lactobacillus sakei* LT-13, constituted a novel bifunctional amino acid racemase that exhibited multiple substrate specificity (7). Industrial-scale synthesis of DAAs—key precursors for β-lactam antibiotics (e.g., cephalosporins) and anticancer peptides (e.g., plitidepsin)—relies heavily on racemases with operational robustness under process conditions (8, 9). For example, the study of glutamate racemase may prove valuable in developing novel antibiotics, as interfering with peptidoglycan biosynthesis is detrimental to bacterial survival (10). These intriguing researches in the area of DAAs are inspiring researchers, including ourselves, to discover new amino acid racemases and uncover novel roles for DAAs in bacteria.

DAAs play a crucial role in microbial physiology as they serve as key constituents of peptidoglycan (11, 12). Peptidoglycan is the building block of the peptidoglycan layer, an indispensable component of the bacterial cell wall. This layer not only determines the cell morphology but also endows bacteria with resistance to osmotic rupture (13). The presence of d-Asp or d-Ser in peptidoglycan structure (14, 15) confers tolerance to certain bactericidal agents such as vancomycin. For example, vancomycin targets the peptidoglycan synthesis process in bacteria. The incorporation of d-Asp or d-Ser into the peptidoglycan structure can interfere with vancomycin’s binding to its target, thereby reducing the drug’s effectiveness and allowing the bacteria to withstand its bactericidal effects (9, 16–18). Additionally, they also form part of microbial secondary metabolites, such as cyclosporin A (19), gramicidin S (20), actinomycin D, and bacitracin (21, 22). Non-canonical DAAs (NCDAAs) (23) are predominantly generated by broad-spectrum amino acid racemases, yet their biological functions remain far from being comprehensively elucidated, and only a few discoveries have been made so far. NCDAAs participate in the process of cell wall remodeling within *Vibrio cholerae* (24, 25), and they can inhibit biofilm formation and trigger biofilm disassembly (26–28). Recent work has shown that d-Arg might be employed for an interspecies altruistic cooperation mechanism so as to assist the multiplication of *Vibrios* within harsh polymicrobial surroundings (29). Consequently, bacteria seem to generate DAAs as a survival strategy when adjusting to environmental changes. Recent studies reveal archaea (particularly *Candidatus Bathyarchaeota* and *Lokiarchaeota*) in deep marine sediments drive DAA metabolism through diverse racemases (AlaR, AspR, BAR), d-serine ammonia-lyase (DSD1), and peptidases (M19, Pab87), converting DAAs to l-amino acids/α-keto acids and hydrolyzing muropeptides. Metagenomic and transcriptomic evidence confirms their global biogeochemical role in organic matter mineralization and carbon/nitrogen cycling (30). On the other hand, DAAs have been found to have extensive applications across diverse domains, including food, cosmetics, and pharmaceuticals. In the food industry, dipeptides or short peptides derived from DAAs are utilized as high-intensity, safe, and calorie-free sweeteners (31). In the realm of cosmetics, DAAs play a pivotal role in formulating moisturizers, hair conditioners, and hair dyes (32). Within the pharmaceutical sector, DAAs and their derivatives serve as crucial intermediates in the synthesis of antibacterial and antiviral agents, as well as antiparasitic drugs (33–35).

Given the critical roles of DAAs in microbial physiology, biomedicine, and the food industry, amino acid racemases and their encoding strains are indispensable in both basic research and biotechnological applications.

To date, amino acid racemases have been extensively studied in multiple bacterial species like *Escherichia coli*, *Bacillus subtilis* (5), *Pseudomonas putida* (1), *Thermococcus litoralis* (36)*, Thermus thermophilus* (37), *Thermococcus kodakarensis* (38)*, Thermotoga maritima* (39), *Thermoanaerobacter tengcongensis* (40), *Pyrococcus horikoshii* OT-3 (41), and *Lactobacillus sakei* (7). Members of the order *Flavobacteriales* are keystone taxa in marine and hypersaline ecosystems, accounting for up to 20% of microbial communities in coastal sediments. Their ecological success is attributed to versatile metabolic capabilities, including the degradation of complex polysaccharides and recalcitrant organic matter (42). Notably, recent metagenomic studies revealed that *Flavobacteriales* genomes are enriched with genes encoding DAA metabolic pathways, particularly in strains inhabiting high-salinity niches (43). This suggests that DAAs, generated via racemization, may play underappreciated roles in osmoregulation and biofilm dynamics under extreme conditions. In hypersaline environments, bacteria face dual challenges of ionic stress and oxidative damage. DAAs are known to enhance peptidoglycan crosslinking, thereby reinforcing cell wall integrity against osmotic lysis (25). However, conventional racemases from non-halophiles often lose activity at high salt concentrations, limiting their utility in halophilic systems. The genomic detection of putative racemases in *Flavobacteriales* implies evolutionary innovations that balance catalytic efficiency with halotolerance—a trait critical for survival in marine sediments and industrial brines. Beyond ecological relevance, halophilic racemases hold biotechnological promise. For instance, β-lactam antibiotic synthesis requires DAAs (e.g., d-Lys) under alkaline conditions (pH 8.0–9.0) (44), yet most commercial racemases suffer from pH instability or substrate promiscuity. A racemase from *Flavobacteriales* could address these limitations by leveraging native adaptations to high pH and salinity. Furthermore, the incorporation of NCDAAs into peptidoglycan is an emerging strategy to combat vancomycin-resistant pathogens (45), necessitating enzymes capable of producing diverse DAAs with high stereoselectivity. Despite the ecological and industrial implications, no amino acid racemase has been functionally characterized in *Flavobacteriales*. Furthermore, the increasing demand for enantiopure DAAs in semisynthetic antibiotics (e.g., cefprozil) and anticancer peptides (e.g., plitidepsin) underscores the urgency to discover robust racemases. Therefore, current research is focused on developing new amino acid racemases with broader substrate specificity, higher activity, and better stability to meet diverse reaction needs.

In this study, we found a novel strain DA487^T^, a representative of the novel family “*Halocolaceae*” of the order *Flavobacteriales*. To characterize strain DA487^T^, a polyphasic approach integrating phylogenetic, genomic, and phenotypic analyses was employed. Through genome analysis of strain DA487^T^, candidate genes involved in the DAA metabolic pathway, with the amino acid racemase gene included, were identified. Subsequently, the racemase gene of strain DA487^T^ was subjected to cloning and expression in *Escherichia coli* BL21(DE3), followed by the characterization of its enzymatic properties *in vitro*. Using molecular docking and site-directed mutagenesis, we constructed several racemase variants to assess their impact on catalytic performance. Our work not only aids in revealing the specific working mechanisms of the racemase in the DA487^T^ strain but also provides a theoretical basis for further optimizing the enzyme’s application in industrial production. Additionally, through in-depth analysis of these variants, we can gain a more comprehensive understanding of the relationship between the structure and function of racemase, offering new avenues for designing and engineering high-efficiency catalysts in the future. Further investigation into the molecular mechanisms and enzymatic properties of amino acid racemase in *Flavobacterium* can expand our understanding of this enzyme’s functionality across different bacterial taxa.

## MATERIALS AND METHODS

### Isolation and cultivation of strain DA487^T^

Strain DA487^T^ was isolated from a sediment sample collected from the Dongying Salt Farm (37°36′ N, 118°41′ E), Shandong, China, in April 2022. The sterile natural seawater was employed to serially dilute the sediment sample. Thereafter, 100 µL of each dilution as an aliquot was plated onto 1/10 Marine agar 2216 (MA, BD) (46). Strain DA487^T^ was isolated and purified on 2216E agar plates. For routine culturing, it was placed on 2216E agar medium at a temperature interval of 28°C to 30°C. Storage was carried out at −80°C in 2216E with 20% glycerol (vol/vol) added.

#### Morphological, physiological, and biochemical characteristics

The characteristics related to the physiology and biochemistry of strain DA487^T^ were assessed. All detailed methodological steps were performed following the protocols established in previous studies (47–49).

#### The 16S rRNA gene and genome sequencing and phylogenetic analysis

Sanger sequencing, with the employment of universal primers 27F and 1492R, enabled the attainment of the 16S rRNA gene sequence of strain DA487^T^ (50). Subsequently, alignment analysis was performed by means of the EzBioCloud server (http://www.ezbiocloud.net/) and the NCBI database (https://blast.ncbi.nlm.nih.gov/Blast.cgi). Based on 37 16S rRNA gene sequences, the phylogenetic tree was preliminarily reconstructed through IQ-TREE (version 1.6.12) in the TVMe + I + G4 mode. In an attempt to gain a clearer understanding of the phylogenetic location of the novel isolates, a sturdy phylogenetic tree was rebuilt. This reconstruction was accomplished by employing FastTree with its preset parameters and IQ-TREE with the GTR + CAT model (51). To assess tree topologies, bootstrap analysis was carried out with 1,000 replications. The visualization of phylogenetic trees was achieved through the utilization of Tree Visualization By One Table (52). The subgroup designations were validated as one cluster exhibited monophyly within two phylogenetic trees that were constructed via different programs and the maximum-likelihood (ML) approach (53–55).

Majorbio Bio-pharm Technology Co., Ltd. (based in Shanghai, China) carried out the sequencing of the entire genome of DA487^T^, making use of the Illumina NovaSeq 6000 and PacBio Sequel II platforms. The data quality control software Fastp (version 0.20.0) was utilized to generate the reads (56). For the evaluation of genome completeness and contamination, CheckM (57) was applied. Subsequently, a total of 270 genomes, which exhibited a completeness level exceeding 95% and a contamination level below 5%, were picked out for the purpose of phylogenomic analysis. By means of GTDB-Tk (version 2.1.1) (58), the concatenated alignment sequences of 120 ubiquitous single-copy proteins were acquired. Thereafter, IQ-TREE with the LG + F + I + G4 (LG model incorporating empirical base frequencies [F], a proportion of invariant sites [I], and a discrete Gamma model with four rate categories [G4]) model was utilized to rebuild the ML phylogenetic tree, which was founded on these amino acid sequences.

#### Genomic analyses

The NCBI Prokaryotic Genome Annotation Pipeline (PGAP), relying on *ab initio* gene prediction algorithms and homology-based methods, was employed to conduct genome annotations of the cultured strains (59). The annotation of the genome sequence was carried out by applying the Rapid Annotations using Subsystems Technology (RAST) (60). The genome data were then stored in NCBI GenBank (accession number GCA_049334165.1). The metabolic pathway was analyzed in detail using KEGG’s BlastKOALA server (https://www.kegg.jp/blastkoala/) (61). Clusters of orthologous groups (COGs) analysis was accomplished using eggNOG-mapper (http://eggnog-mapper.embl.de/) (62, 63). A comparison was made between the 16S rRNA gene sequence and the taxonomically unified 16S rRNA gene database available in EzBioCloud (64) to identify similar species. Afterward, the 16S rRNA gene and genome sequences pertaining to these species were amassed with the intention of being used in subsequent analyses. The RAST server was utilized to perform a re-annotation of the assembled genomes. The values of AAI (average amino acid identity) and POCP (percentage of conserved proteins) were determined by making use of an R script (https://github.com/2015qyliang/POCP) in line with the methods that had been detailed previously (65, 66).

### Generation of expression plasmids pertaining to the wild-type and the variant RacX

PCR was employed to amplify the amino-acid racemase gene RacX, with the genomic DNA of strain DA487^T^ serving as the template and a pair of primers (RacX-F and RacX-R in Table S4) being utilized. The PCR products were purified using a gel extraction kit (Vazyme, China). Then, the linearized pET28a vector digested with *BamH*I and *Xho*I (37°C, overnight) and the target gene fragment (RacX) were seamlessly ligated using homologous recombination technology with Cloning Kit (Vazyme, China) (the mixture was incubated at 37°C for 30 min). The resulting ligation product was immediately transformed into *Escherichia coli* DH5α-competent cells. The plasmid was verified by digestion with restriction enzymes *BamH*I and *Xho*I (NEB, New England Biolabs) and by DNA sequencing.

The DNA polymerase (Vazyme, China) was used for mutating the residues Cys193 to Ser193 (TGC→AGC), Ala79 to Cys79 (GCC→TGC), Asn80 to Ala80 (AAC→GCC), Thr81 to Ala81 (ACC→GCC), Asn121 to Ala121 (AAC→GCC), and Thr124 to Ala124 (ACA→GCA) within the substrate access region of RacX. The PCR amplification yielded the products using primer pairs C193S-F and C193S-R, A79C-F and A79C-R, N80A-F and N80A-R, T81A-F and T81A-R, N121A-F and N121A-R, and T124A-F and T124A-R, as summarized in Table S4. Nucleotide sequencing was performed on the expression plasmids of wild-type and variant RacX by RuiBiotech in Qingdao, China. The constructs bearing the accurate gene sequences were converted into *E. coli* Rosetta (DE3) to conduct enzyme expression, purification, and racemization assay.

#### Expression and purification of recombinant proteins

The constructs were utilized in the generation process of proteins, which were tagged with histidine at both the N-terminal and C-terminal, with *E. coli* Rosetta (DE3) being the host system for such production. The N-/C-terminal His-tag was retained for purification efficiency; control assays confirmed that it does not alter enzyme kinetics. In the context of gene overexpression, 500 mL of LB culture was inoculated with an overnight culture of Rosetta (DE3) cells harboring the constructs. This LB culture was then incubated at 37°C under shaking conditions until the OD_600_ value approximated between 0.4 and 0.6. Subsequently, 0.3 mM isopropyl-β-d-thiogalactopyranoside was added to the culture for induction. It was then shaken vigorously at a speed of 220 rpm for 3 h at 30°C. After that, the induced cells were collected through centrifugation and kept in storage at −80°C.

In the process of protein purification, the harvested cells were first put into 20 mM sodium phosphate buffer (with a pH of 7.4), which also contained 500 mM NaCl and 50 mM imidazole, and then lysed by means of sonication. Next, the lysates obtained were subjected to centrifugation at 12,000 × *g* for 20 min at 4°C, and the supernatant was subjected to immobilized metal affinity column chromatography with the HyPur T Ni-IDA 6FF (His-Tag) PrePacked Gravity Column (Sangon Biotech, China). After loading, Buffer A (consisting of 20 mM sodium phosphate buffer, with a pH of 7.4, and 500 mM NaCl) was supplemented with 80 mM imidazole, which was utilized to carry out the washing of the column, and then was eluted with buffer A containing 300 mM imidazole. The buffer of the eluted protein fractions was replaced with 50 mM Tris-HCl (pH 8.0) containing 10% glycerol using Spectra/Por dialysis membranes (6–8 kD). The determination of protein concentrations was carried out by means of the protein assay dye reagent provided by Bio-Rad. To evaluate the apparent protein purity, purified samples were subjected to sodium dodecyl sulfate-polyacrylamide gel electrophoresis with the application of 12% or 15% gels. The purified protein’s concentration was ascertained by Nanodrop. Subsequently, 50 µL aliquots of the purified protein were rapidly chilled in liquid nitrogen and maintained at −80°C for storage.

#### Racemase activity assays

All the racemase assays were performed with minor modifications according to the protocol described in references (23, 67). To begin with, a mixture was made up in a final volume of 50 µL, which consisted of 50 mM Tris-HCl (with a pH of 7.5) and l-amino acid at different concentrations, and 2.5 µg of racemase (wild-type and variant RacX protein). To confirm that our endpoint measurements reflected the initial velocity, the linearity of the reaction was assessed by quantifying product formation at time intervals from 0 to 120 min. The reaction rate remained linear (*R*² >0.99) for the first 60 min (Fig. S3). Therefore, a 30-min incubation period, which lies well within this linear phase, was adopted for all routine activity assays and kinetic analyses to guarantee that the recorded activities represent true initial velocities. The reaction was performed for 30 min. Then, reactions were stopped by adding 2 M HCl, and an equal volume of 2 M NaOH was added immediately to neutralize. To clear the inactivated proteins, the samples underwent a spinning process at 14,000 rpm for 10 min. Subsequently, the amount of DAA produced was determined by joining 50 µL of the extract with 100 µL of a reaction containing 200 mM Tris-HCl, pH 8.0, 1.0 U/mL DAA oxidase (DAAO) (Yuanye Biotech, Shanghai, China), 5 U/mL horseradish peroxidase, 0.1 mg/mL 4-aminoantipyrine, and 0.1 mg/mL TOOS (*N*-ethyl-*N*-(2-hydroxy-3-sulfopropyl)-3-methylaniline, sodium salt). l-Amino acid oxidases (Yuanye Biotech, Shanghai, China) deaminate amino acids to α-keto acids and generate hydrogen peroxide (68). Thus, the method for measuring the isomerization of DAAs to l-amino acids is similar to that for measuring the isomerization of l-amino acids to DAAs (69). The only differences are that DAAO is replaced with l-amino acid oxidase, and the substrate l-amino acid is replaced with DAA. Meanwhile, positive and negative control experiments were conducted, and the results demonstrated that this method could be applied to the detection of the conversion of DAAs to l-amino acids. The reaction was incubated at 37°C for a period of 45 min and subsequently inactivated with 2 M HCl, ultimately yielding a colorimetric product that can be measured at a wavelength of 550 nm. The above racemase assay reactions were all subjected to triplicate experiments.

#### Substrate specificity

Racemase activities and substrate specificities of RacX were investigated by employing d-Lys and a variety of l-amino acids, namely l-Ala, l-Val, l-Leu, l-Ile, l-Ser, l-Thr, l-Gln, l-Asn, l-Met, l-Lys, l-Arg, l-Phe, l-Trp, l-Tyr, l-His, l-Pro, and l-Orn as substrates. The assay mixture (50 µL) for RacX (2.5 µg) contained 5 mM amino acid and 50 mM Tris-HCl, pH 7.5. To measure the racemase activity with respect to different amino acids, reaction mixtures were placed at 37°C and incubated for 30 min. In the subsequent experiment, the reaction products from the previous step were incubated in 200 mM Tris-HCl, pH 8.0 at 37°C for 45 min or 1 h and subsequently inactivated with 2 M HCl, ultimately yielding a colorimetric product that can be measured at a wavelength of 550 nm. Triplicate experiments were performed for each of the aforementioned reactions.

#### Effect of pH on enzyme activity

The examination of the impact of pH on RacX was conducted through the measurement of its racemase activities toward 5 mM l-Lys under standard assay circumstances. The assay mixture (with a volume of 150 µL) consisted of 5 mM l-Lys, 7.5 µg of purified enzyme, and 50 mM buffer at different pH levels (5.0–10.5). For this assay, the following buffers were used: sodium acetate for pH 5.0–6.0, sodium phosphate for pH 6.0–7.0, Tris-HCl for pH 7.0–9.0, and CAPSO for pH 10.5. Incubation of the reaction mixtures was carried out at 37°C for 30 min. Each of the above reactions had three parallel experiments arranged.

#### Effect of temperature on enzyme activity

To investigate the impact of temperature on RacX’s enzyme activity, the racemase activity toward l-Lys was measured. The assay mixture (with a volume of 150 µL) was composed of 5 mM l-Lys, 7.5 µg purified enzyme, and 50 mM Tris-HCl (pH 7.5). Incubation of the reaction mixtures was carried out at temperatures varying from 25°C to 70°C, and RacX’s activity was determined after 30 min of incubation. In the determination of the above reactions, three parallel experiments were established.

#### Effect of metal ions on enzyme activity

The effect of various metal ions on the activity of the racemase enzyme was assessed under optimal reaction conditions (37°C, pH 7.5). Metal salts—namely ZnSO₄, CuSO₄, CaCl₂, MgCl₂, FeSO₄, CdCl₂, KCl, NiCl₂, and CoCl₂—were added to the enzyme reaction system at a final concentration of 1 mM. All assays were conducted in triplicate to ensure experimental reproducibility. The control group, which contained no metal ions, was assigned a relative activity value of 100%.

#### Thermal inactivation assay

Thermal inactivation assays were performed by pre-incubating RacX (0.5 mg/mL) in 50 mM Tris-HCl (pH 7.5) at 45–70°C. Aliquots were withdrawn at 20-min intervals, rapidly cooled on ice, and residual activity toward l-Lys (5 mM) was measured under standard conditions (37°C, pH 7.5).

#### Kinetic analyses

The kinetic parameters of RacX were additionally ascertained through the utilization of l-AA (0.5 mM–100 mM) with purified enzyme (2.5 µg). For the measurement of the reactions, three parallel experiments were arranged. At 37°C, the RacX racemase reaction was executed in a reaction mixture (with a volume of 50 µL) consisting of 50 mM Tris-HCl (pH 7.5), 2.5 µg of RacX, and the substrate. The reaction duration was set at 30 min, with the substrate concentrations for the racemase reaction being 1, 2, 5, 10, 20, 40, or 100 mM. Kinetic parameters (*V*_max_ and *K*_*m*_ values) for the reaction were determined using a Michaelis-Menten plot.

## RESULTS AND DISCUSSION

### Collection of strain DA487^T^

**Fig 1:**
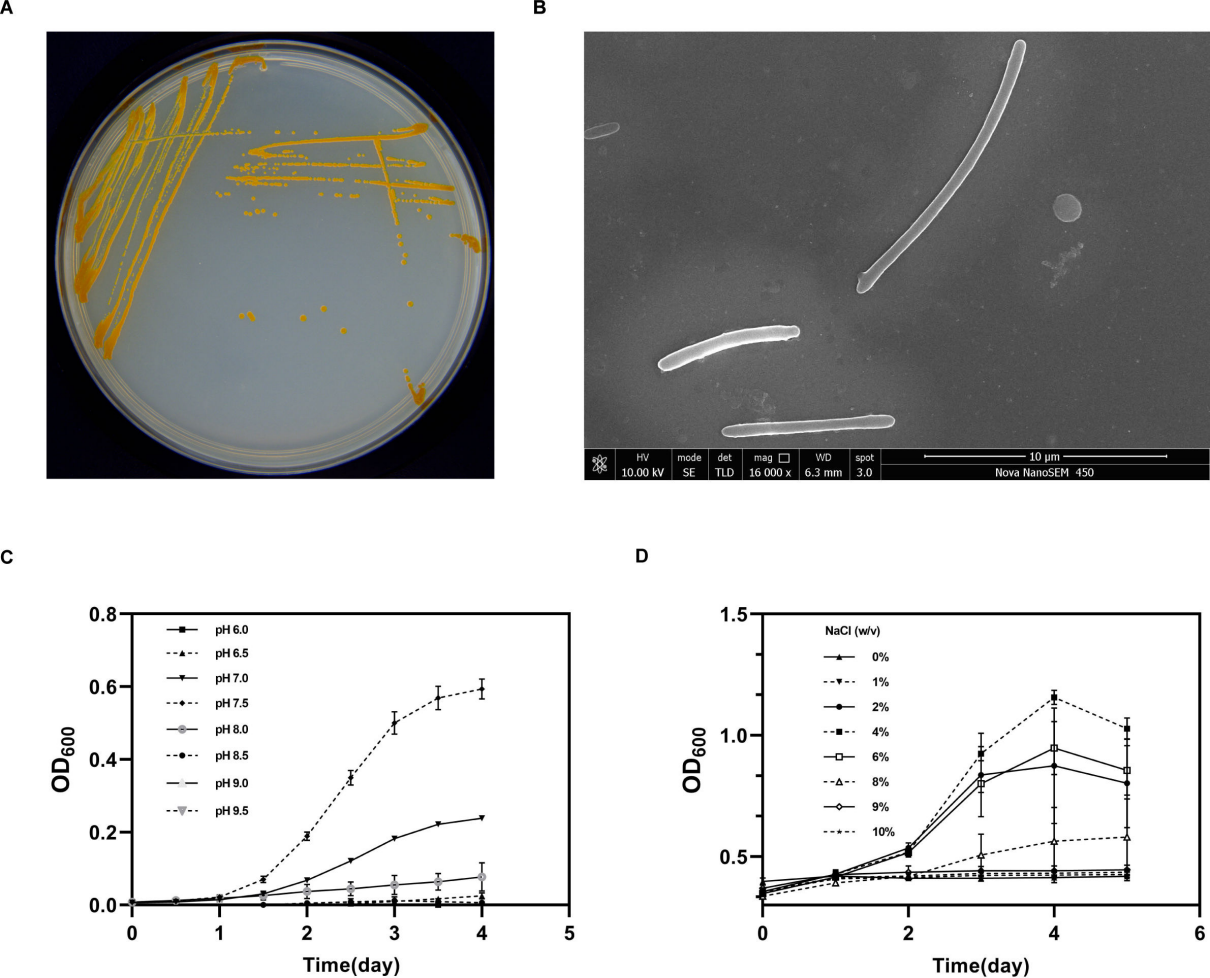
Displaying the phenotypic traits of strain DA487^T^. (**A**) The colony’s morphological attributes. (**B**) The morphological properties of an individual cell. (**C and D**) Growth curves of strain DA487^T^ with different pH conditions (**C**) and varying NaCl concentrations (**D**). Every group of experiments underwent triple replication.

Among 120 candidate strains isolated from hypersaline sediments, DA487^T^ was selected for its unique 16S rRNA phylogeny (88.6% identity to closest relatives) and gene signatures of DAA metabolism (e.g., RacX homologs). On marine agar 2216, the colony of strain DA487^T^ was round, possessing a moist surface and regular edges, and it showed orange after incubation for 5 d (Fig. 1A). By means of a scanning electron microscope and a light microscope, the morphology and internal structure of strain DA487^T^ were inspected, as can be seen in Fig. 1B.

#### Phenotypic characteristics of strain DA487^T^

The temperature span in which strain DA487^T^ on marine agar 2216 was from 28°C to 40°C with the optimum at 35°C. Strain DA487^T^ could grow at pH 6.0–pH 9.5 with the optimum at pH 7.5 (Fig. 1C), and its salt tolerance concentration range was from 0% to 8% with the optimum at 4% (Fig. 1D). The DA487^T^ cells were typically rod-shaped, with widths ranging from 0.5 to 0.7 µm and lengths ranging from 2.5 to 10 µm. Moreover, through Gram staining, strain DA487^T^ was Gram-negative. A juxtaposition of the phenotypic idiosyncrasies of strain DA487^T^ and the type species of other families in the taxonomic order of *Flavobacteriales* was manifested in Table S1.

#### Chemotaxonomic characteristics of strain DA487^T^

MK-7 was identified as the respiratory quinone in strain DA487^T^, which was consistent with that of its closely related taxa, specifically members of the *Owenweeksiaceae* (70) and *Croceimicrobium hydrocarbonivorans* A20-9^T^ (71); however, MK-6 was the predominant quinone in *Owenweeksia hongkongensis* DSM 17368^T^ (72), another member of the family *Owenweeksiaceae*, as shown in Table S1. The fatty acid distribution pattern of DA487^T^ and that of the phylogenetically associated members within the family *Owenweeksiaceae* are given in Table S2. The polar lipid profiles of strain DA487^T^ included phosphatidylethanolamine (PE), phosphatidylcholine (PC), and two unidentified lipids (L) (Table S2). An unidentified lipid (L) present in strain DA487^T^ was not identified in the close relatives.

#### Strain DA487^T^ belongs to a novel family of the order *Flavobacteriales*

**Fig 2:**
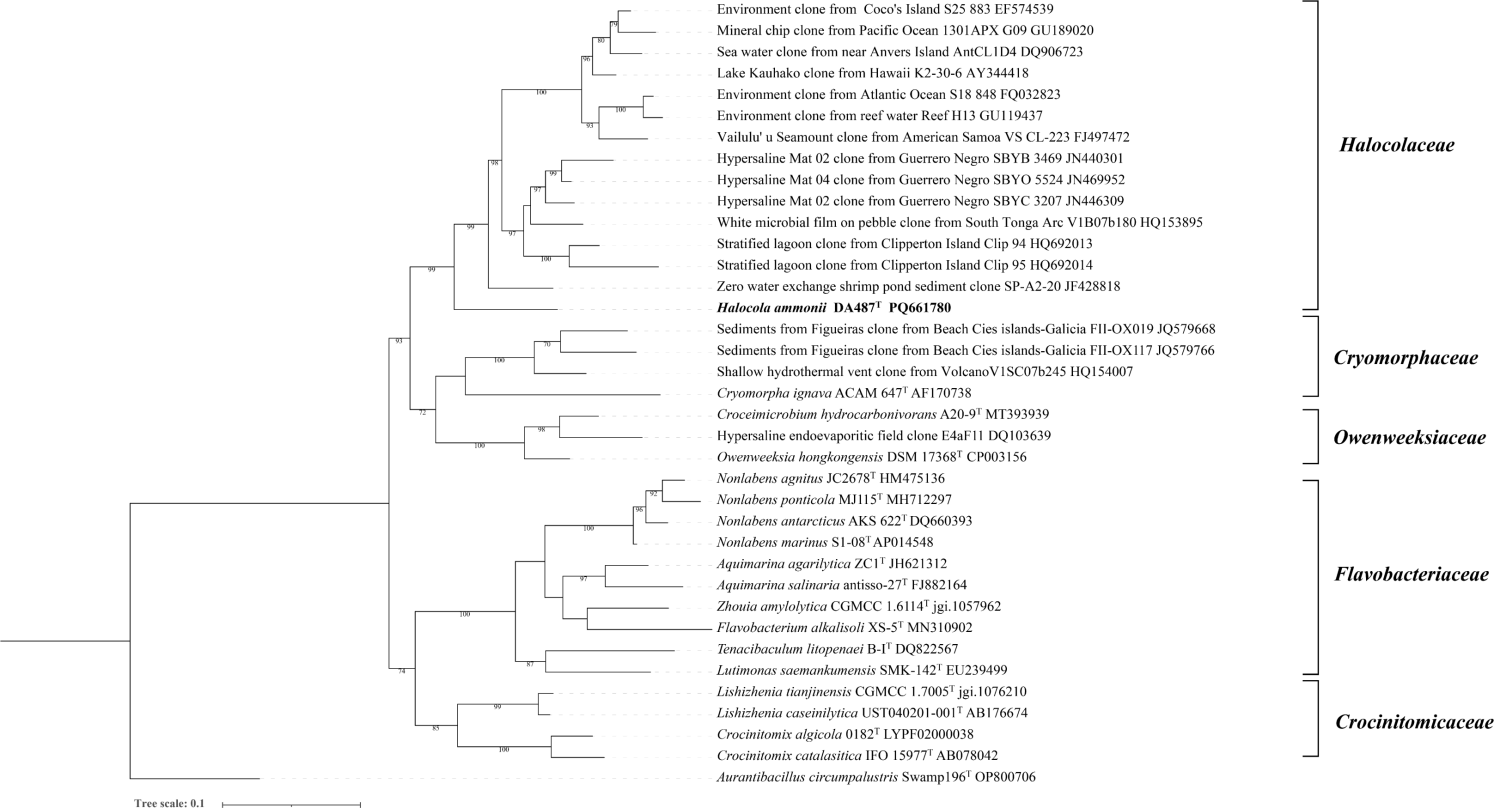
ML tree depicting phylogenetic connections derived from the 16S rRNA gene sequences of DA487^T^ and closely associated species within the order *Flavobacteriales*, comprising both cultured representatives and environmental clones. *Aurantibacillus circumpalustris* Swamp196^T^ was used as the outgroup. At the branch nodes, bootstrap values higher than 70% (from 1,000 replicates) are displayed. Bar, 0.1 substitutions for each nucleotide position.

To discover related species, the whole 16S rRNA gene sequence (1,487 bp) of DA487^T^ was utilized to perform similarity-focused searches against the taxonomically amalgamated 16S rRNA gene database in EzBioCloud. The 16S rRNA gene sequence of DA487^T^ showed 88.62% and 87.70% identity values with that of *C. hydrocarbonivorans* A20-9^T^ and *O. hongkongensis* DSM 17368^T^, respectively. The 16S rRNA gene sequences of closely allied species were amassed from the EzBioCloud and employed to assemble the phylogenetic tree using the ML method. In accordance with the phylogenetic trees constructed from 16S rRNA gene sequences (Fig. 2 and Fig. S1), strain DA487^T^ was closely related to *C. hydrocarbonivorans* and *O. hongkongensis* and was in a separate clade within the order *Flavobacteriales*.

**Fig 3:**
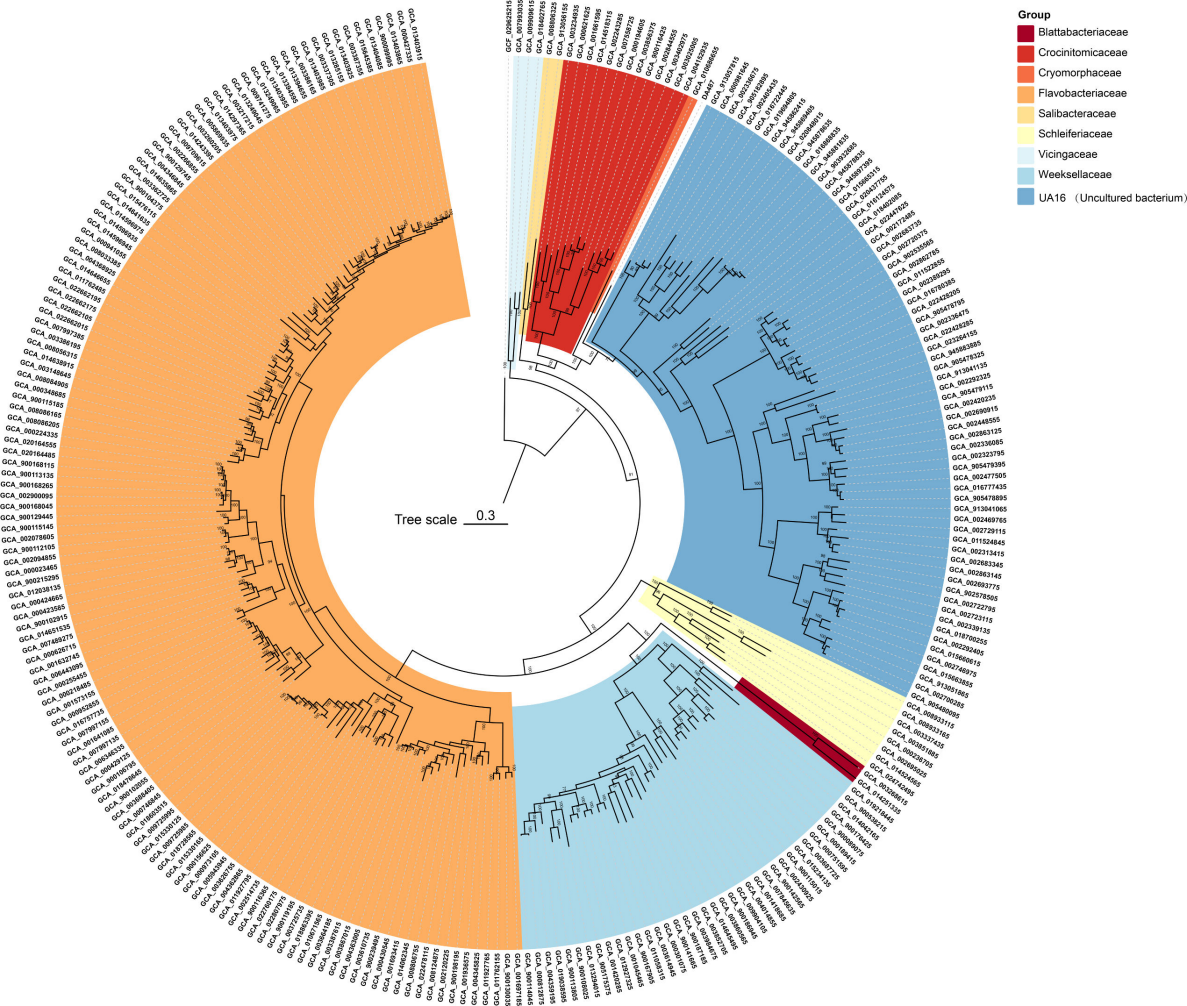
Phylogenomics tree showing phylogenetic relationships based on the protein sequences of DA487^T^ and closely related members of the order *Flavobacteriales*. Bootstrap values are based on 1,000 replicates; only bootstraps exceeding 70% are shown. Bar, genetic distance of 0.3. *Aurantibacillus circumpalustris* Swamp196^T^ was used as an outgroup.

Furthermore, the IQ-TREE analysis, which was grounded on 120 ubiquitous single copy proteins, indicated that strain DA487^T^ and certain uncultured clones constituted a distinct branch within the order *Flavobacteriales,* accompanied by high bootstrap values. This finding buttressed the conclusion that it symbolizes a novel family (Fig. 3). The mean values of amino acid identities in relation to strain DA487^T^ compared with those of *C. hydrocarbonivorans* A20-9^T^ and *O. hongkongensis* DSM 17368^T^ were 57.1% and 57.5%, respectively (Fig. S2) (AAI). These values fall within the threshold range of a new family (45%–65%) (73–75). The POCP values calculated between strain DA487^T^ and *C. hydrocarbonivorans* A20-9 ^T^ as well as *O. hongkongensis* DSM 17368 ^T^ were 33.1% and 33.5%, respectively (Fig. S2). All these values supported that strain DA487 ^T^ should be classified into a novel family (65). The 16S rRNA gene and genome sequences have been deposited in GenBank under accession numbers PQ661780 and GCA_049334165.1, respectively.

#### Genomic features and phylogenomics of strain DA487^T^

Strain DA487^T^ had a genome consisting of a single chromosome, which had a total length of 3,787,011 bp and a G + C content of 44.6% (as shown in Table S1). The results of the NCBI PGAP predicted the existence of 3,208 protein-coding genes and 42 RNA genes, including 1 16S rRNA, 1 5S rRNA, 1 23S rRNA, 3 ncRNA, and 36 tRNA genes.

**Fig 4:**
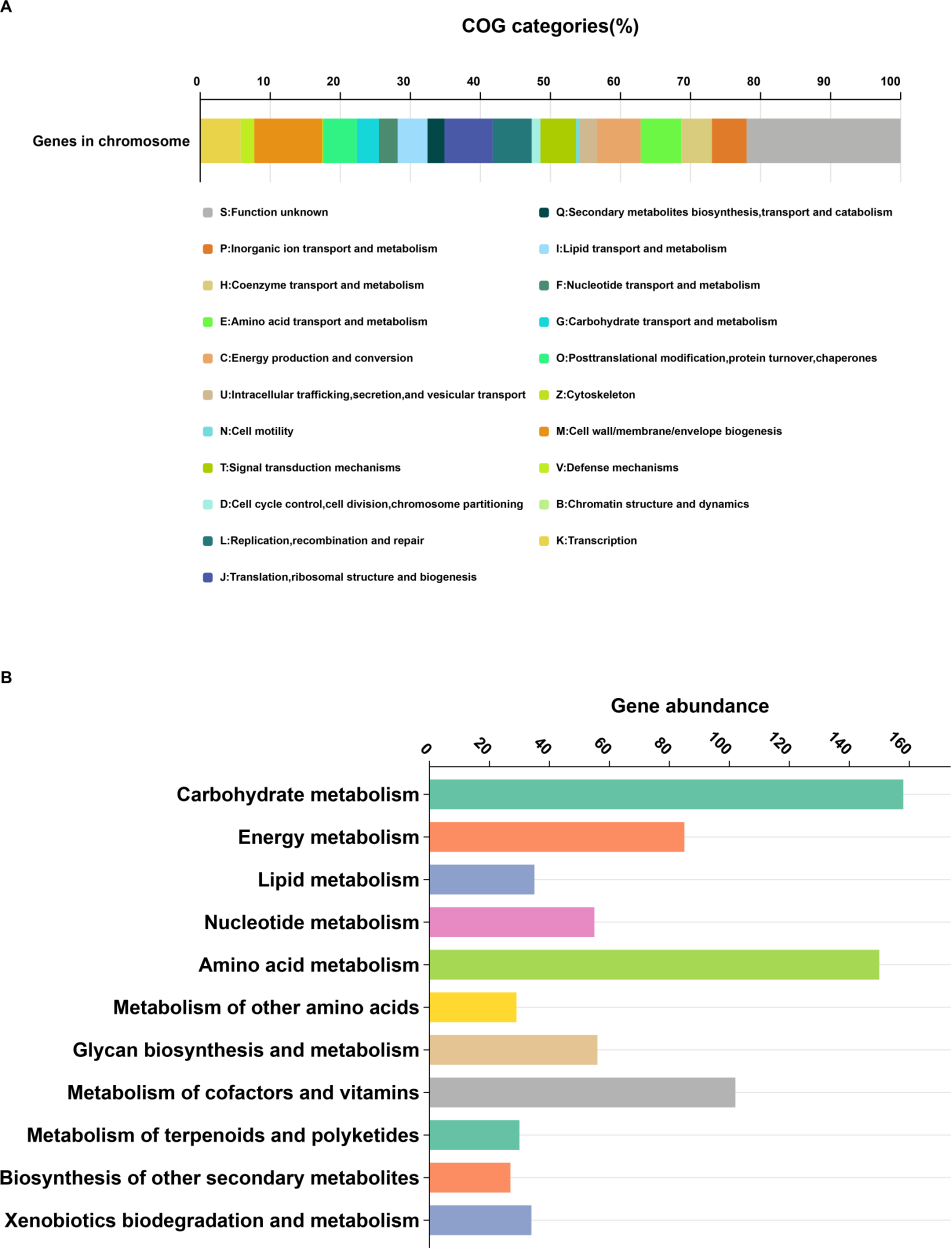
Genome features of the *H. ammonii* DA487^T^ chromosome. (**A**) Distribution of COG categories for chromosome genes. (**B**) Analysis of the metabolic pathways of DA487^T^ from KEGG.

For a more profound comprehension of the function of DA487^T^, the CDSs on the chromosome were meticulously annotated through the employment of the COGs assignment. Altogether, 2,320 (65.7%) CDSs were sorted into COG families that encompassed 21 categories (as depicted in Fig. 4A). The results revealed that “M: Cell wall/membrane/envelope biogenesis” (with 225 genes), “J: Translation, ribosomal structure and biogenesis” (with 160 genes), “C: Energy production and conversion” (with 145 genes), “K: Transcription” (with 134 genes), and “E: Amino acid transport and metabolism” (with 134 genes) were the most enriched functional categories compared with others on the chromosome. A high proportion of CDSs (48.8%) was poorly characterized, including “S: Functional unknown” (510 genes) and “no homologs identified” (1212 genes). KEGG’s BlastKOALA service was utilized to analyze metabolic pathways (Fig. 4B). The results of strain DA487^T^ indicated a robust metabolic capacity, particularly in carbohydrate and amino acid metabolism, with the presence of complete pathways such as glycolysis, pyruvate oxidation, the citrate cycle, gluconeogenesis, PRPP biosynthesis, and the pentose phosphate pathway. Notably, strain DA487^T^ harbors multiple genes (K01775, K01921, K01425, K01925, K01776, K01778, K25316, K21898) related to DAA metabolism (Table S3), which suggests a potential for active DAA biosynthesis or degradation. Among them, amino acid racemases are responsible for catalyzing the interconversion between DAAs and l-amino acids, and this interconversion is crucial for various biological processes, including protein synthesis, bacterial cell wall synthesis, and metabolic regulation. Given the extensive genetic foundation of amino acid metabolism, further investigation into the specific amino acid racemases present in DA487^T^ could unveil novel enzymatic activities and provide insights into the strain’s ecological niche and biotechnological potential, potentially leveraging its capabilities in industrial applications such as asymmetric synthesis or pharmaceuticals.

### Identification of a gene candidate associated with DAA metabolism

According to the extensive metabolic profile of strain DA487^T^, which includes multiple genes related to DAA metabolism, the identification of specific racemases is pivotal for understanding the metabolic capabilities and potential applications of this strain. Derived from the comparison of the putative metabolic pathways constructed with the aid of the KEGG Automatic annotation server (76), several specificity racemases such as alanine racemase (77–81), glutamate racemase (82, 83), and ornithine racemase, as well as a putative broad-specificity racemase (84) that present in the DAA metabolic pathway of strain DA487^T^ and responsible for catalyzing the l-to-d racemization of amino acids (Table S3). Apparently, the synthesis of d-Arg, d-Lys, and d-Orn is achieved by a broad-spectrum racemase (RacX) from *Bacillus subtilis* (5). Yet, the purposes of these DAAs have not been determined. Recognizing the substantial roles that DAAs play in diverse biological processes, such as microbial interactions, antibiotic production, and potential therapeutic applications, it is crucial to understand the function of RacX in strain DA487^T^. Thus, to examine the enzyme function of RacX in strain DA487^T^, the cloning and overexpression of the *RacX* gene were carried out in *E. coli*.

#### Purification and structure features of RacX amino acid racemases

**Fig 5:**
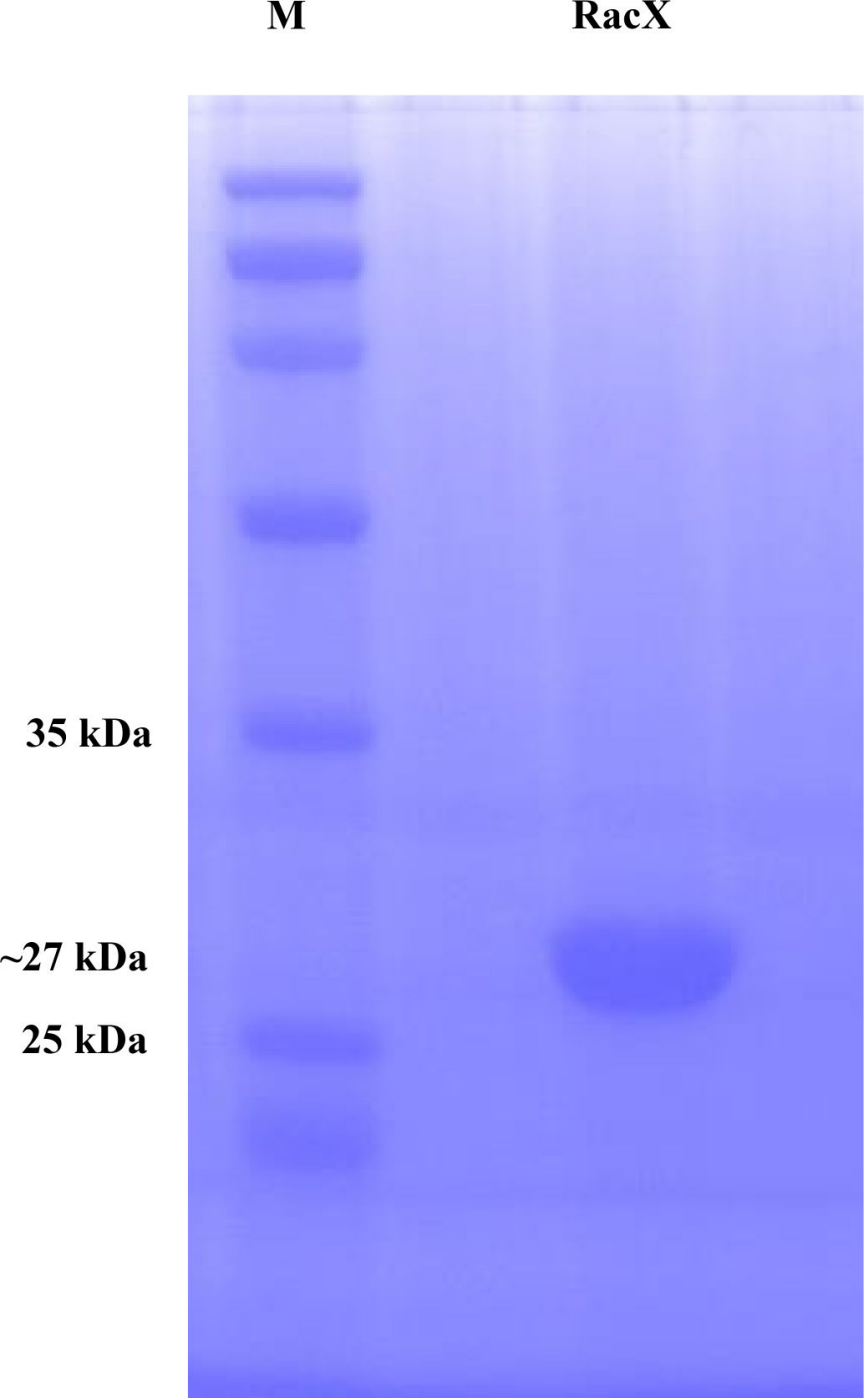
Purification of the recombinant RacX protein. Two micrograms of the RacX protein sample was applied, and the gel was stained with Coomassie brilliant blue. M, molecular weight marker.

**Fig 6:**
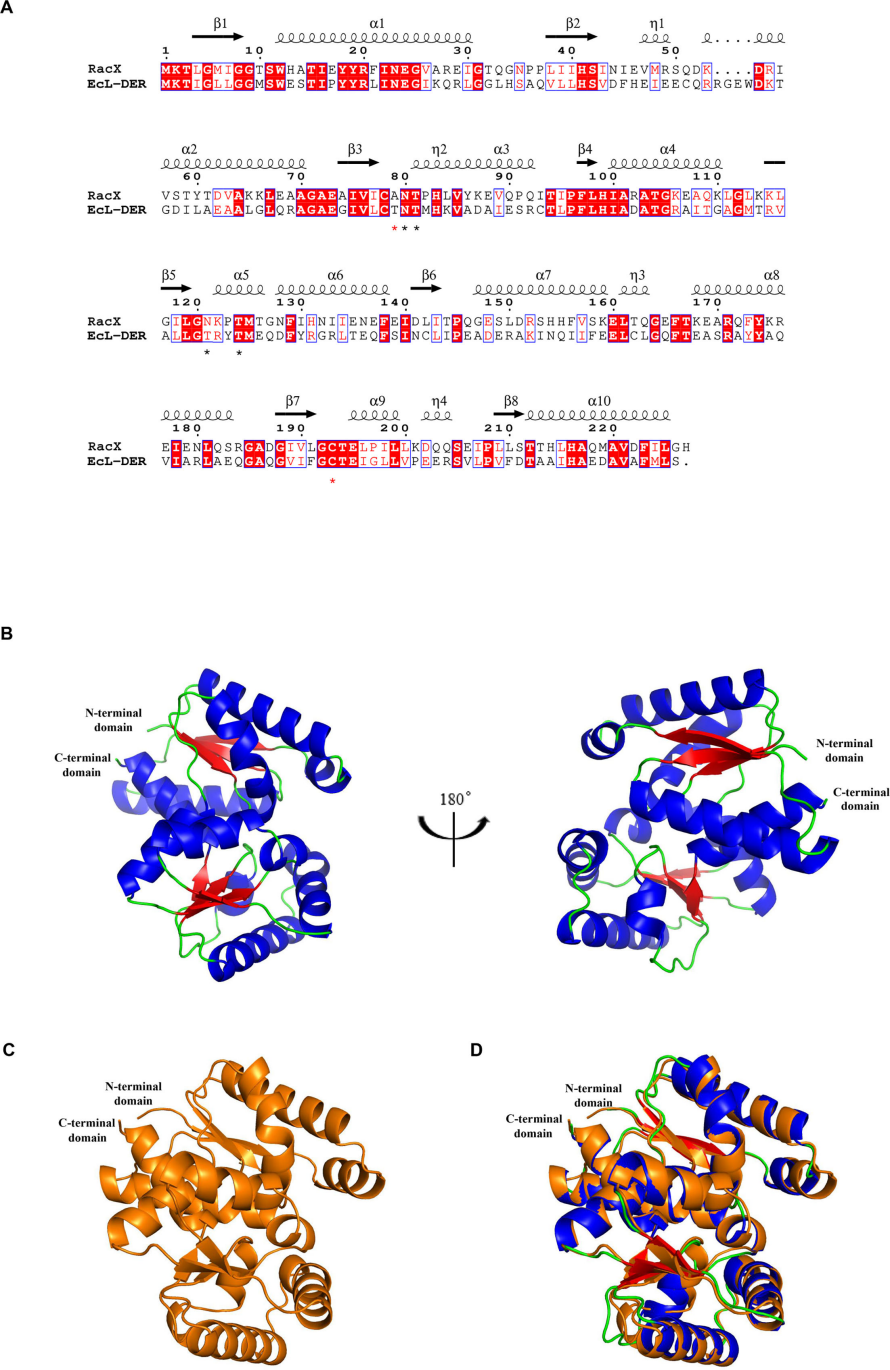
Comparative structural analysis of RacX and EcL-DER. (**A**) Sequence alignment of RacX and *E. coli* l-aspartate/glutamate racemase (EcL-DER, PDB: 5ELL) was performed using Clustal Omega. Catalytic residues (Ala79, Cys193 in RacX; Thr83, Cys197 in EcL-DER) are marked with red asterisks. Key residues (Asn80, Thr81, Asn121, and Thr124 in RacX) are marked with black asterisks. Secondary structure elements (α-helices: cylinders; β-strands: arrows) are annotated based on RacX’s AlphaFold-predicted structure. (**B**) Cartoon representation of RacX generated by AlphaFold2 (pTM = 0.94). (**C**) Crystal structure of EcL-DER (orange, PDB: 5ELL). (**D**) Structural superimposition demonstrating high conservation of active site geometry (RMSD = 0.75 Å over 218 Cα atoms).

Using Ni-NTA affinity column chromatography, RacX overproduced in *E. coli* was purified until it reached homogeneity. The RacX subunit with a C-terminal and an N-terminal hexa-histidine tag had a molecular weight of approximately 27 kDa, as inferred from the amino acid sequence (Fig. 5). To elucidate the putative function of the gene product of RacX from strain DA487T, we analyzed the amino acid sequence and tertiary structural characteristics of RacX. It was shown that the overall amino acid configuration of RacX bore a remarkable similarity to those of EcL-DER (PDB ID: 5ELL) (with 35.4% amino acid sequence identity) (Fig. 6A) via SWISS-MODEL on ExPASy web server (https://swissmodel.expasy.org). RacX conformational modeling (pTM = 0.94, pTM means the predicted template modeling, and a pTM score above 0.5 means the overall predicted fold for the complex might be similar to the true structure) (Fig. 6B) was done via Alphafold server (https://alphafoldserver.com/) and PyMOL 3.0. AlphaFold-predicted models of RacX (pTM = 0.94) showed high structural similarity to EcL-DER (RMSD = 0.75 Å, Fig. 6D), consistent with previous crystallographic studies of the EcL-DER active site (85, 86). While these predictions suggest conserved catalytic mechanisms (e.g., Cys193/Thr81 pairing), direct experimental validation of RacX’s tertiary structure is needed to confirm residue roles. A comparison was made between the structural model of the RacX subunit of this study and the subunit structure of the l-aspartate/glutamate-specific racemase from *Escherichia coli* (EcL-DER) (Fig. 6C) (85) using PyMOL 3.0, which suggested that the root mean square deviation (RMSD) value was 0.754 (Å) (Fig. 6D) (RMSD values are always >0, and an RMSD value of 0 means it is perfectly aligned). To sum up, the amino acid sequence and the tertiary conformation of RacX are remarkably similar to those of EcL-DER. We predict that there may be identical active catalytic sites in the conserved domains of both (see subsequent work).

#### Substrate specificity of RacX

RacX’s racemase activities were analyzed through the utilization of 17 diverse l-amino acids, with one non-proteinogenic amino acid being present: Ala, Thr, Pro, Gln, Ser, Asn, Met, Val, Leu, Ile, Phe, His, Arg, Tyr, Lys, Trp, and Orn. RacX catalyzed the racemization of 15 amino acids. Based on catalytic efficiency (*k_cat_/K_m_*), l-Lysine was the preferred substrate for RacX (*k*_cat_*/K_m_* = 151.2 s⁻¹mM⁻¹). The enzyme also exhibited high efficiency (*k*_cat_*/K_m_* > 100 s⁻¹mM⁻¹) for l-Trp, l-Asn, l-Gln, and l-Ala (see Table 1), suggesting its potential roles in metabolic pathways involving these compounds. These observations highlight the potential utility of RacX in biotechnological applications targeting specific amino acids and indicate areas for further research to enhance its substrate range and catalytic efficiency. The absence of activity toward l-Met and l-His may be indicative of structural constraints or specific binding affinities that restrict its catalytic scope (87), warranting further structural and kinetic studies to fully elucidate these mechanisms. Overall, these findings provide valuable insights into the catalytic capabilities and limitations of RacX, guiding future enzyme engineering efforts and applications in diverse biochemical processes.

**TABLE 1:** Substrate specificity of RacX racemase toward different l-amino acids^*a*^^,^^*b*^

Substrate	*K_m_* (mM)	*k*_cat_ (s^−1^)	*k*_cat_*/K_m_* (s^−1^mM^−1^)
l-Lys	1.6 ± 0.5	241.8 ± 12.4	**151.2**
l-Trp	0.3 ± 0.2	44.2 ± 1.0	**140.6**
l-Thr	1.1 ± 0.7	40.9 ± 1.1	36.9
l-Met	ND	ND	ND
l-Val	23.9 ± 3.1	323.0 ± 20.3	13.5
l-Leu	0.6 ± 0.2	57.8 ± 5.8	96.9
l-Ile	17.1 ± 5.8	140.0 ± 17.4	8.2
l-Ala	1.0 ± 0.5	113.3 ± 15.6	**113.7**
l-His	ND	ND	ND
l-Tyr	0.6 ± 0.2	56.1 ± 3.1	94.5
l-Arg	0.7 ± 0.1	42.1 ± 6.7	60.0
l-Asn	0.6 ± 0.5	84.1 ± 13.7	**141.9**
l-Ser	3.4 ± 0.5	71.1 ± 15.7	20.7
l-Orn	0.9 ± 0.5	54.6 ± 6.1	59.7
l-Phe	2.5 ± 1.4	63.3 ± 14.6	25.1
l-Gln	0.5 ± 0.1	56.7 ± 3.6	**121.6**
l-Pro	0.6 ± 0.2	51.1 ± 9.5	82.6

^*a*^ ND, no data.

^*b*^ Bold terms indicate *k_cat_/K_m_* > 100 s⁻¹mM⁻¹.

#### Effects of temperature and pH on the racemase activity of RacX

**Fig 7:**
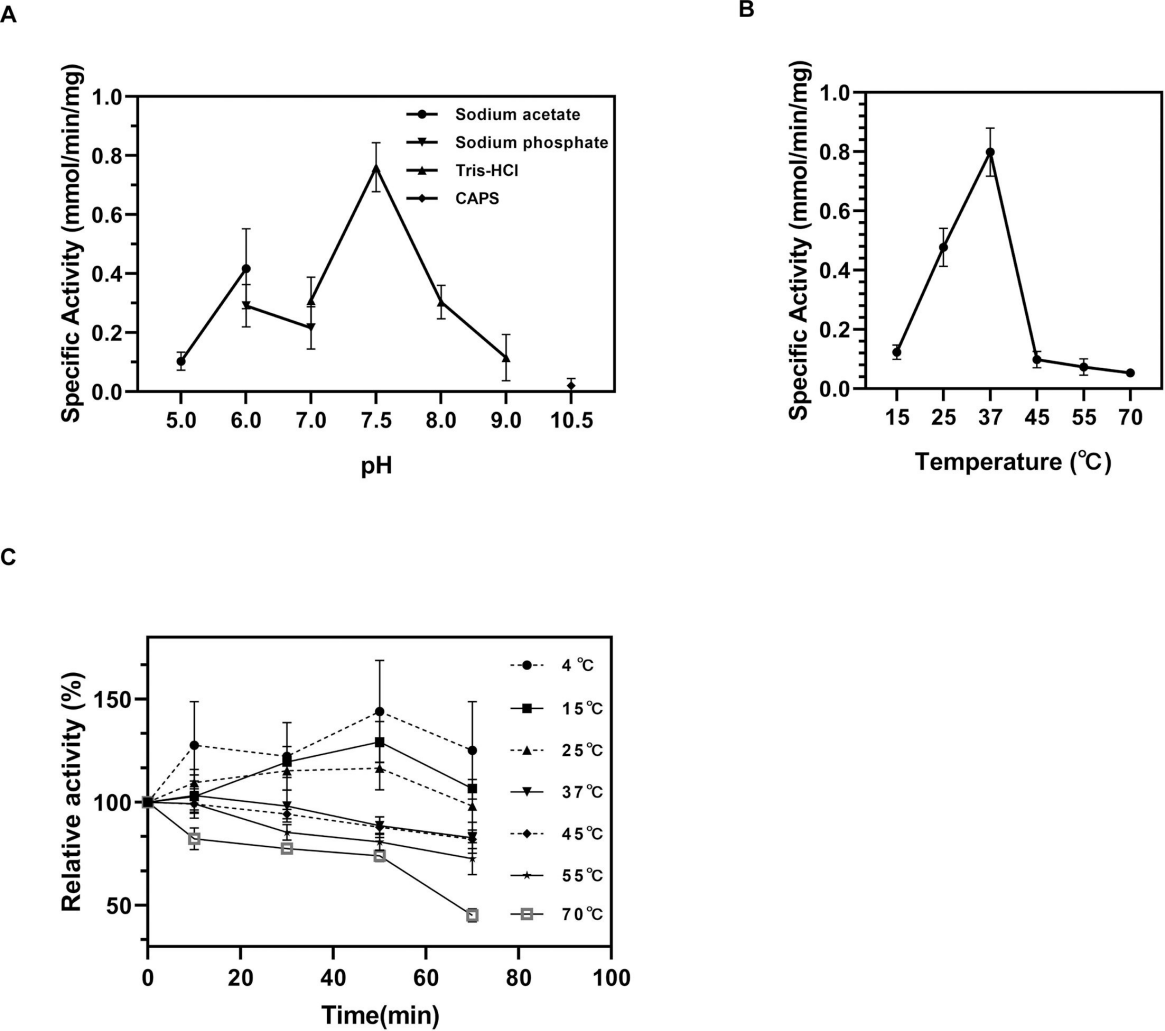
Effects of environmental factors on RacX activity. (**A**) pH-activity profile measured in 50 mM buffers: acetate (pH 5.0–6.0), phosphate (pH 6.0–7.0), Tris-HCl (pH 7.0–9.0), and CAPSO (pH 10.5). (**B**) Temperature optimum assay (25°C–70°C) in 50 mM Tris-HCl (pH 7.5). Activity declines sharply above 45°C due to thermal inactivation, as shown in panel **C**. (**C**) Thermal inactivation kinetics. Residual activity (%) was determined at the indicated time points (0–70 min). Panel C demonstrates the rapid thermal inactivation of RacX at temperatures above 45°C, explaining the sharp activity decline observed in panel **B**. Error bars represent SD from triplicate experiments. Each group of experiments was replicated three times.

At 37°C, the impact of pH on the racemase activity of RacX for l-Lys was examined over a variety of pH values from 5.0 to 10.5. The activity grew as the pH went up from 5.0 to 10.5, with the optimum activity found at pH 7.5. The activity was extremely low at pH 5.0 (Fig. 7A). The influence of temperature on the racemase activity of RacX with regard to l-Lys was investigated at diverse temperatures spanning from 15°C to 70°C. The activity increased from 15°C to 37°C, reaching its peak at 37°C. Subsequently, the activity decreased as the temperature rose from 37°C to 70°C, and the enzyme exhibited an almost zero enzymatic reaction rate between 60°C and 70°C (Fig. 7B). This trend aligns with similar studies on amino acid racemases, which typically exhibit optimal activity within physiological pH and temperature ranges (80, 88). Minimal activity was observed at pH 5.0, likely due to destabilizing electrostatic interactions affecting the enzyme’s active site or substrate binding (89). Conversely, the fact that the highest activity is observed at pH 7.5 suggests that this condition stabilizes the enzyme’s conformation and optimizes its substrate affinity. The temperature profile revealed that the activity increased from 15°C to 37°C, which is consistent with the enzyme’s role in biological systems. However, a sharp decline in activity at 45°C indicated its thermal sensitivity, with a complete loss of activity at 60°C–70°C, which is a common feature of many thermolabile enzymes (90).

Thermal inactivation kinetics revealed a strong temperature dependence of RacX stability. The enzyme retained >80% activity after 1 h at 55°C but rapidly lost activity above 70°C (Fig. 7C).

The moderate thermostability of RacX ($T_{\text{1/2}}^{\text{55°C}}=\;70\;min$) positions it between typical mesophilic and thermophilic racemases. While outperforming mesophilic homologs like *Bacillus subtilis* RacX (5), RacX is less robust than hyperthermophilic counterparts such as *Thermococcus kodakarensis* TK1211 (*$T_{1/2}^{\text{90°C}}\;>10\;h$*) (38). This intermediate stability may reflect evolutionary adaptation to its native niche-hypersaline sediments experiencing diurnal thermal fluctuations. Overall, RacX exhibited pH- and temperature-dependent activity profiles, with maximal activity at pH 7.5 and 37°C, aligning with its putative physiological role in moderately thermophilic environments.

#### Effect of metal ions on the racemase activity of RacX

The effect of several metal ions (Ni^2+^, Zn^2+^, K^+^, Co^2+^, Ca^2+^, Cu^2+^, Cd^2+^, Fe^2+^, and Mg^2+^) on the activity of RacX was examined using l-Lys as a substrate, as detailed in Table 2. Some metal ions, such as Cu^2+^, Zn^2+^, and Fe^2+^, significantly enhanced the enzyme activity. However, the other metal ions reduced the enzymatic activity to varying degrees. The metal ion profile revealed a complex interplay between RacX and various cations. The significant enhancement of activity by Cu²^+^ suggests a potential allosteric or structural stabilization role, rather than a destructive interaction with the catalytic cysteine. Although Cu²^+^ is often an inhibitor of cysteine-dependent enzymes, its role as an activator or structural cofactor in specific contexts is documented and aligns with the known ability of metal ions to modulate enzyme activity through diverse mechanisms (91–93). Conversely, the strong inhibition observed with Cd²^+^, a metal ion with high affinity for thiol groups (94), provides independent biochemical evidence consistent with the presence of a cysteine residue critical for catalysis, as identified in our structural and mutagenesis studies. This spectrum of effects underscores the uniqueness of the RacX active site microenvironment.

**TABLE 2:** Effect of several metal ions on the activity of RacX

Metal ion	Concentration (mM)	Relative activity (%)
None	0	100
Ni^2+^	1	58.0 ± 14.6
Zn^2+^	1	132.6 ± 14.6
Mg^2+^	1	82.2 ± 6.7
Co^2+^	1	54.4 ± 19.8
Cd^2+^	1	27.4 ± 5.1
Ca^2+^	1	37.5 ± 5.2
Fe^2+^	1	181.4 ± 17.6
Cu^2+^	1	317.2 ± 25.3
K^+^	1	96.6 ± 5.9

#### Kinetic analysis of RacX

The determination of the kinetic parameters of RacX racemase activity with respect to l-Lys was carried out, yielding a *K*_*m*_ value of 1.6 ± 0.5 mM and a *k*_cat_ value of 241.8 ± 12.4 s^−1^, as shown in Table 3. The *K_m_* value of 1.6 mM indicated moderate substrate affinity of RacX for l-Lys, comparable to values reported for other racemases and amino acid-metabolizing enzymes. Based on existing research, the *K*_*m*_ values of racemases from *Escherichia coli* (5), *Bacillus subtilis*, *Thermococcus kodakarensis* (38), *Pyrococcus horikoshii* OT-3 (41), *Lactobacillus* (7, 95), *Thermococcus litoralis* (36), *Pseudomonas putida* KT2440 (1), and other strains for their respective l-AA substrates ranged from approximately 2.2 mM to 169.0 mM. Thus, RacX racemase appears to have a higher affinity for l-Lys than some other racemases have for their respective substrates. The *k*_cat_ value of 241.8 s^−1^ suggested that RacX racemase was highly efficient in catalyzing the racemization of l-Lys. When comparing it to other well-characterized racemases, such as the proline racemase from *Pseudomonas putida* with a *k*_cat_ of around 70 s^−1^ (1) (Table S5), RacX racemase demonstrated a significantly higher catalytic efficiency. The *k*_cat_/*K*_*m*_ ratio, which represents the enzyme’s overall catalytic efficiency, can be calculated as 151.2 s^−1^mM^−1^. This relatively high value suggested that RacX racemase was both efficient and specific in processing l-Lys, further supporting its potential biological relevance in l-amino acid metabolism.

**TABLE 3:** Kinetic parameters of RacX variant proteins^*a*^

Enzyme	Substrate	*K_m_* (mM)	*k*_cat_ (s^−1^)	*k*_cat_*/K_m_* (s^−1^mM^−1^)	*K*eq
RacX	l-Lys	1.6 ± 0.5	241.8 ± 12.4	151.2	8.5
RacX	d-Lys	25.3 ± 10.6	291.2 ± 50.5	11.5	
RacX_A79C_	l-Lys	25.1 ± 6.1	403.7 ± 40.6	16.1	0.6
RacX_A79C_	d-Lys	40.0 ± 13.7	662.7 ± 109.3	16.6	
RacX_C193S_	l-Lys	14.6 ± 2.7	596.7 ± 39.2	40.9	–^*b*^
RacX_N80A_	l-Lys	3.7 ± 2.1	128.5 ± 17.6	34.7	–
RacX_T81A_	l-Lys	7.1 ± 1.8	205.9 ± 15.1	29.0	–
RacX_N121A_	l-Lys	8.7 ± 1.6	198.0 ± 11.0	22.8	–
RacX_T124A_	l-Lys	6.2 ± 0.8	591.3 ± 20.9	95.4	–

^*a*^ N80A, T81A, N121A, and T124A variants were assayed under standard conditions (5 mM l-Lys, 37°C, pH 7.5).

^*b*^ –, no data.

These results indicated that RacX racemase exhibited moderate substrate affinity for l-Lys and a high catalytic efficiency and was similar to other well-characterized racemases.

#### Biotechnological implications for pharmaceutical applications

DAAs serve as critical chiral building blocks in synthetic antibiotics and anticancer agents. For instance, d-Lysine, a preferred substrate of RacX (*k*_cat_*/K_m_* = 151.2 s^−1^mM^−1^), is an essential precursor in semisynthetic cephalosporins (e.g., cefprozil), where its incorporation enhances β-lactam ring stability against bacterial hydrolases (9). Similarly, non-proteinogenic DAAs like d-Arg, which RacX efficiently produces (Table 1), are increasingly employed in tumor-targeting peptides (e.g., plinabulin) to evade proteolytic degradation *in vivo* (96, 97). RacX’s broad substrate promiscuity positions it as a candidate for generating DAA mixtures. However, industrial production of enantiopure DAAs typically requires coupling RacX with enantioselective downstream processes (e.g., dynamic kinetic resolution) (98), as racemases inherently yield racemic products. Notably, the enzyme’s moderate thermolability ($T_{\text{1/2}}^{\text{55°C}}\;=\;70\;min$) allows rapid heat inactivation to terminate reactions, minimizing unwanted epimerization—a feature critical for high-purity DAA production (99).

Emerging applications in combating antimicrobial resistance could also leverage RacX’s activity. DAAs like d-Asp and d-Ser are integrated into peptidoglycan precursors to disrupt vancomycin binding, offering a strategy to potentiate existing antibiotics (9, 16–18). RacX’s ability to synthesize these NCDAAs (Table 1) under mild conditions may enable co-administration therapies to restore drug efficacy against resistant pathogens. However, enzymatic incorporation of NCDAAs into peptidoglycan remains mechanistically complex and requires further validation.

#### Identification of RacX catalytic and key residues

**Fig 8:**
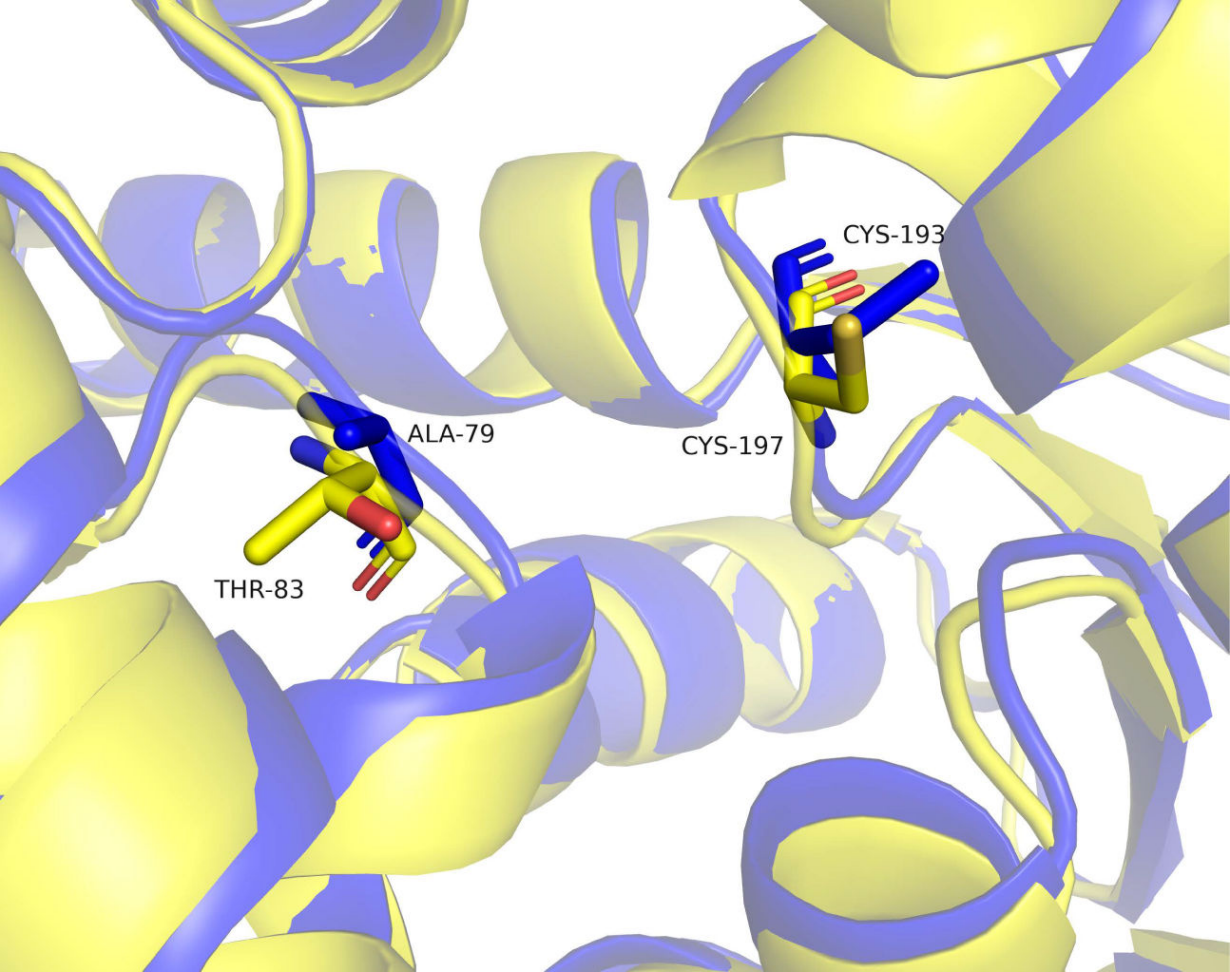
Structural alignment of RacX (blue) and EcL-DER (yellow) active sites. Key residues are shown as sticks: RaX (Ala79—blue and Cys193—orange) and EcL-DER (Thr83—red and Cys197—green).

Through amino acid sequence alignment analysis and tertiary conformation analysis (Fig. 6A and 8), we observed that the residue, Ala79, in RacX aligned with the residue, Thr83, in EcL-DER. It has been reported that EcL-DER showed racemase activity when l-Asp and l-Glu were used as the substrates, while no racemase activity was observed when d-Asp and d-Glu were used as the substrates. This may be due to the fact that the amino acid at position 83 is Thr, not Cys, in EcL-DER (85). However, wild-type RacX (Ala79) possesses significant bidirectional activity, catalyzing both l-Lys→d-Lys and d-Lys→l-Lys conversions (Table 3). This functional divergence from the strictly l-specific EcL-DER highlights a key difference in stereochemical control despite partial structural conservation. We then conducted a comprehensive kinetic characterization of both wild-type and A79C variant RacX. The A79C variant exhibited a 1.4-fold enhancement in catalytic efficiency (*k*_cat_*/K_m_*) for the reverse reaction (d-Lys → l-Lys) compared to wild type (25.6 vs. 17.8 s⁻¹mM⁻¹), while its forward reaction efficiency decreased approximately ninefold (Table 3). At the catalytic site, the exchange of Ala79 with cysteine, as these results suggest, primarily rebalances the enzyme’s catalytic equilibrium rather than creating *de novo* activity—the A79C mutation reduced the thermodynamic preference for d-Lys production (*K*eq = 8.5 for WT vs. *K*eq = 0.6 for A79C). It is crucial to clarify that enzymes, as catalysts, do not alter the fundamental thermodynamic equilibrium between free l-amino acids and DAAs in solution, for which ΔG° =0 and *K*eq, intrinsic = 1 (100). The calculated equilibrium constants (*K*eq = (*k*_cat_/*K_m_*)_l__→__d_/(*k*_cat_/*K_m_*)_d__→__l_) are apparent values (*K*eq,app) derived from the Haldane relationship (101). They reflect a strong kinetic preference or bias inherent to the enzyme-substrate complex (102). The wild-type enzyme’s active site is optimized to catalyze the l- to d- conversion more efficiently, leading to a *K*eq,app of 8.5. The A79C mutation alters the active site architecture, thereby rebalancing the kinetic parameters for the forward and reverse reactions and resulting in a Keq,app of 0.63. This represents a change in the enzyme’s catalytic bias (103), not a violation of thermodynamic principles. The Ph1733 protein, a structural homolog of PhAspR derived from *Pyrococcus horikoshii* OT3, harbors an alanine substitution at the residue position equivalent to Cys82 in PhAspR (Ala82) and demonstrates complete loss of racemase activity toward both l- and d-enantiomers of aspartate and glutamate (104). Notably, despite the conserved Ala residue at the equivalent position in both RacX and Ph1733 (Ala79 in RcaX, Ala81 in Ph1733), significant functional divergence is observed between these enzymes. This contrast underscores that Ala79 in RacX functions as a modulator of enantiomeric preference within an active racemase scaffold, rather than as an absolute determinant of activity. However, the specific mechanisms require further experimental exploration and analysis.

Furthermore, Cys193 corresponds to the catalytic active site Cys197 in Ec-DER (Fig. 8). When Cys197 was replaced by serine, the variant showed no racemase activity, indicating that Cys197 might function as a nucleophile in the enzyme catalysis (85). To determine whether the Cys193 residue also affects the function of RacX, we constructed variant protein C193S (Table S4) and performed racemization assays. As expected, the enzyme activity of C193S was markedly reduced (Table 3). The C197S substitution increased catalytic turnover (*k_cat_*) but reduced substrate affinity (*K_m_*), suggesting that Cys197 is more involved in substrate binding than in catalysis.

**Fig 9:**
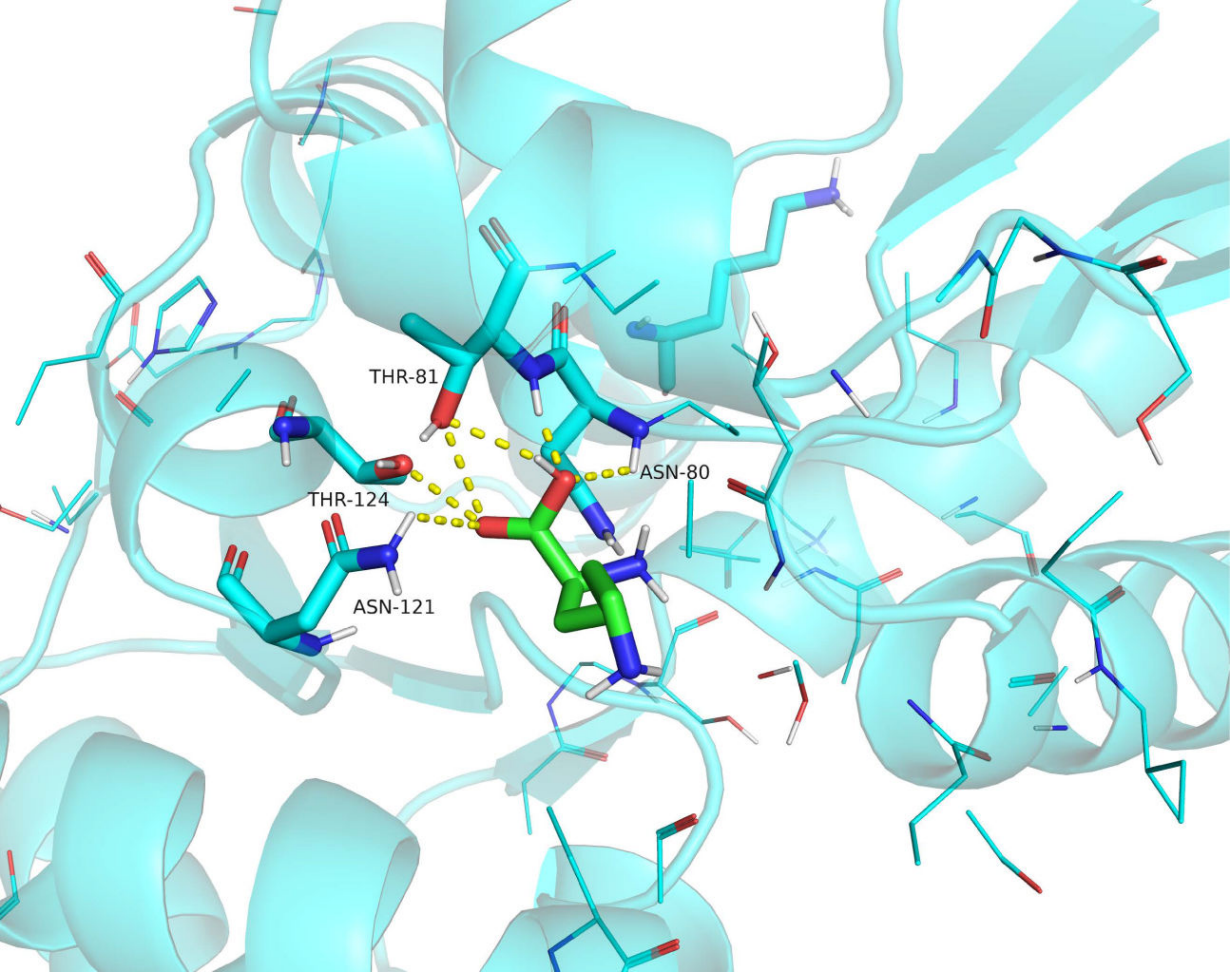
Molecular docking of l-Lys into RacX active site. Global view of l-Lys (red-blue sticks) docked into RacX-binding pocket. Hydrogen-bond interactions (dashed lines) between l-Lys and Asn80/Thr81/Asn121/Thr124. Docking performed using AutoDock Vina.

Molecular docking studies integrating AlphaFold-predicted structural models with PyMOL-based analyses were performed to identify additional functionally critical sites in RacX through enzyme-substrate interaction profiling (Fig. 9), we identified four potential key residues of the RacX protein domain: Asn80, Thr81, Asn121, and Thr124. We then introduced amino acid substitutions at these three residues to create the N80A, T81A, N121A, and T124A variants (Table S4) and were assayed under standard conditions (5 mM l-Lys, 37°C, pH 7.5). The results showed that the enzyme activities of the variants were significantly lower compared to the wild-type RacX (Table 3). This indicates that these residues, Asn80, Thr81, Asn121, and Thr124, are crucial for the enzyme activity of RacX. Comparative kinetic analysis revealed distinct perturbation patterns among the variants. The N80A, T81A, and N121A variants exhibited 1.3-fold to 4.4-fold increased *K_m_* values accompanied by 15%–85% reductions in both *k*_cat_ and catalytic efficiency (*k*_cat_*/K_m_*) relative to wild-type enzyme. This dual impairment in substrate affinity and catalytic capacity suggests structural perturbations affecting (i) the integrity of substrate-binding pocket interactions, (ii) proton relay networks essential for catalysis, or (iii) conformational dynamics required for catalytic cycling. Strikingly, the T124A variant displayed a unique kinetic profile characterized by 2.9-fold elevated *K_m_* and 37% decreased *k*_cat_*/K_m_*, yet paradoxically demonstrated 1.4-fold enhanced *k_cat_*. This decoupling phenomenon implies that T124 primarily modulates substrate binding through steric constraints, while its substitution may enhance the electrostatic microenvironment of the catalytic core. These proposed mechanisms await further interrogation via complementary structural biology approaches coupled with functional assays to establish definitive mechanistic linkages.

### Description of the novel taxa

#### Description of *Halocola* gen. nov

*Halocola* (Ha.lo.co′ la. From Gr. fem. n. hals, meaning sea, saline, or salt; and cola, an N. L. suffix masc./fem., meaning an inhabitant of; thus, *Halocola* refers to a microbe of the sea). Cells are Gram-negative, rod-shaped or bent rod-shaped, non-motile, and facultative anaerobes. Cells can grow with DAAs. The major fatty acids are iso-C15:0 and anteiso-C15:0. The primary respiratory quinone is MK-7. The polar lipid profile mainly consists of PE, PC, and two unidentified lipids (L), as well as an unidentified lipid (L).

#### Description of *Halocola ammonii* sp. nov

*Halocola ammonii* (a.m.mo′ni.i. N.L. n. ammonium, ammonia). Apart from those specified for the genus, it also demonstrates the following traits. Cells are 0.5–0.7 µm in diameter and 2.5–10 µm long. No flagella are found around the cells. Colonies assume a circular shape, are smooth, possess an orange color, and measure 2 mm in diameter. Their edges are entire and transparent following a 3-day incubation period at 30°C. Growth occurs at 28°C−42°C (optimum 28°C−30°C) and pH 8.5–9.5 (optimum pH 9.0) with 2.0% (wt/vol)−8.0% (wt/vol) NaCl (optimum 4%). These cells do not hydrolyze Tween 60, Tween 80, cellulose, alginate, or agar. As per the API ZYM kits, cells registered as positive for alkaline phosphatase and esterase (C4) trypsase, cystine arylamidase, esterase lipase (C8), leucine arylaminase, valine arylaminase, chymotrypsin, naphthol-AS-BI-phosphohydrolase, and acid phosphatase, but negative for lipase (C14), α- and β-galactosidase, β-glucuronidase, α- and β-glucosidase, N-acetyl-β-glucosaminidase, α-mannosidase, and α-fucosidase.

The type strain, DA487^T^ (=KCTC 102213^T^= MCCC 1H01450^T^), was isolated from marine sediment from Dongying Salt Farm, China. For the type strain, the DNA G + C content is 44.6 mol%.

#### Description of *Halocolaceae* fam. nov

*Halocolaceae* (Ha.lo.co.la.ce′ae. N.L. fem. n. *Halocola* type genus of the family; L. suff. -aceae ending to denote a family; N.L. fem. pl. n. *Halocolaceae,* the family of the genus *Halocola*. gen. nov). The characteristics described are similar to those of the genus *Halocola. Halocola* gen. nov. is set as the type genus.

### Conclusion

This study delineates *Halocola ammonii* DA487^T^ as the founding member of the novel family *Halocolaceae*, expanding the phylogenetic diversity of halophilic *Flavobacteriales*. The broad-specificity racemase RacX represents a paradigm shift in enzymatic DAA production, combining high catalytic efficiency (*k*_cat_*/K_m_* = 151.2 s^−1^mM^−1^) with unique bidirectional activity mediated by Cys193 and Ala79. The biotechnological promise of RacX lies in this unique combination of broad substrate promiscuity and exceptionally high catalytic efficiency for specific, high-value amino acids. Its outstanding efficiency positions it as an ideal candidate for the industrial-scale production of d-Lysine, a critical chiral building block for β-lactam antibiotics (e.g., cefprozil). Furthermore, this broad specificity is not a dilution of its application potential but a significant asset, conferring operational flexibility for the synthesis of other DAAs like d-Arg or d-Trp without changing the enzymatic biocatalyst. Its innate halotolerance and stability under alkaline conditions (pH 7.0–8.5) make it particularly suited for prevalent industrial processes.

Future work will therefore not merely address enantiomeric purity through strategies like fusion with enantioselective enzymes, but will also focus on sharpening its selectivity or shifting its catalytic equilibrium (as demonstrated with the A79C variant) for specific synthetic goals, such as dynamic kinetic resolution (DKR) processes.

While RacX’s moderate thermostability ($T_{\text{1/2}}^{\text{55°C}}\;=\;70\;min$) limits high-temperature applications, rational engineering targeting flexible loops (e.g., N80P/T124P substitutions) could enhance robustness without compromising activity—a strategy successfully applied to *Thermus thermophilus* glutamate racemase. Combining homology modeling with mutagenesis, we propose that Asn80/Thr81/Asn121/Thr124 contribute to RacX’s substrate binding, a hypothesis supported by conservation with EcL-DER. Future crystallographic studies are essential to refine this blueprint for biocatalyst design.

This work not only enriches our understanding of racemase evolution in understudied taxa but also establishes a framework for developing tailored biocatalysts to meet the growing demand for enantiopure DAAs in pharmaceuticals and agrochemicals.

## Data Availability

Data will be made available on request. The accession numbers in GenBank/EMBL/DDBJ for the 16S rRNA and genomic gene sequence of strain DA487^T^ are PQ661780 and GCA_049334165.1, respectively.

## SUPPLEMENTAL MATERIAL

Supplemental material aem.02015-25-s0001.pdf Tables S1 to S5, Fig. S1 to S5, and *racX* gene sequence.

ASM does not own the copyrights to Supplemental Material that may be linked to, or accessed through, an article. The authors have granted ASM a non-exclusive, world-wide license to publish the Supplemental Material files. Please contact the corresponding author directly for reuse.
