# Supplementary material for: Identification and characterization of RacX, a new broad-specificity amino acid racemase from a novel taxon within the order Flavobacteriales
Source: Appl Environ Microbiol. 2025 Dec 22;92(1):e02015-25. doi: 10.1128/aem.02015-25 (PMC12838360; doi:10.1128/aem.02015-25)
Supplement: Supplemental material — Tables S1 to S5, Fig. S1 to S5, and racX gene sequence. [file aem.02015-25-s0001.pdf]

Identification and Analysis of RacX：a New Broad-Specificity Amino Acid Racemase from a strain of a novel taxon within order *Flavobacteriales*

Li Hu [a], Xin-Yun Tan [a], Yu-Qi Ye [a], Xin-Yu Liu [a], Jing-Yao Wang [a], Yu-Zhu Li [a], Ting-Ran Zhang [a], Zong-Jun Du [a, c], Meng-Qi Ye* [a, b, c]

[a] Marine College, Shandong University, Weihai, Shandong, 264209, PR China.

[b] Shenzhen Research Institute, Shandong University, Shenzhen 518057, PR China

[c] Weihai Research Institute of Industrial Technology of Shandong University, Weihai, 264209, PR China

* Correspondence:

yemengqi@sdu.edu.cn（M.-Q.-Y）

Supplemental Materials

Table S1. Phenotypic properties of *Halocola ammonii* DA487^T in comparison with those of phylogenetically related members of the family *Owenweeksiaceae.*

Strains: 1. *Halocola ammonii* DA487^T; 2. *Croceimicrobium hydrocarbonivorans* A20-9^T; 3. *Owenweeksia hongkongensis* DSM 17368^T. Data for strain DA487^T are from this study; data for strains 2 and 3 are from the references. +, Positive; −, negative; ND, no data.

Characteristics	1	2	3
Isolation	Marine	Marine	Marine
Colony color	Orange	Orange	Orange
NaCl range (%, w/v)	1.0–8.0	1.0-8.0	1.0-7.5
pH optimum	7.0	7.5	6.0-8.0
Temp. Range (°C)	15-40	10-40	4-37
**Enzyme activity:**			
Oxidase	-	-	+
Trypsin	+	+	ND
Catalase	-	+	+
**Hydrolysis of:**			
Tween 20	+	+	+
Tween 80	-	+	+
Starch	+	-	-
**Utilization of:**			
D-maltose	-	ND	-
Sucrose	-	-	-
Turanose	+	ND	-
Lactose	-	ND	-
D-fructose	-	+	+
Fucose	+	ND	-
**Major quinone**	MK-7	MK-7	MK-6
**Genome size (bp)**	3,787,011	4,365,762	4,622,888
**DNA G + C content (mol %)**	44.6	43.2	39.6±0.5

Table S2. Cellular fatty acids of *Halocola ammonii* DA487^T compared with those of phylogenetically related members of the family *Owenweeksiaceae.*

Strains: 1. *Halocola ammonii* DA487^T; 2. *Croceimicrobium hydrocarbonivorans* A20-9^T; 3. *Owenweeksia hongkongensis* DSM 17368^T. Values given are percentages of total fatty acids. Data for strain DA487^T are from this study; data for strains 2 and 3 are from Liu et al. (2021). Major fatty acid components (>10.0%) are highlighted in bold type. ND, Not detected; TR, trace amount (<1.0%). *Summed features are groups of two or three fatty acids that cannot be separated by GLC using the MIDI system. Summed feature 3 comprised 16:1 w7c and/or 16:1 w6c. Fatty acids were extracted following the standard protocol of the Sherlock Microbial Identification System (MIDI, version 6.1). Subsequent analysis was performed using a gas chromatograph (Model 6890 N, Agilent Technologies), and the identification of fatty acid components was achieved via matching with the TSBA40 database integrated into the Sherlock Microbial Identification System.

Fatty acid	1	2	3
Iso-C14:0	3.0	2.7	TR
C15:0	ND	ND	ND
C16:0	1.8	1.5	2.7
Iso-C15:0	**47.4**	**37.5**	**38.1**
Iso-C15:1 G	4.9	5.3	**12.1**
Anteiso-C15:0	**22.6**	TR	TR
Iso-C16:0	2.8	1.2	1.3
Iso-C17:0	TR	ND	TR
C15:0 2 OH	1.9	TR	2.9
C15:0 3 OH	ND	3.5	ND
C16:0 3 OH	TR	ND	1.4
Iso-C15:0 3 OH	3.1	8.1	2.8
Iso-C16:0 3 OH	1.5	**12.4**	TR
Iso-C17:0 3 OH	3.0	9.9	9.5
Summed Feature 3*	TR	**11.6**	**14.2**

Table S3. The key genes predicted to be involved in DAA catabolism in strain DA487^T.

Gene ID	Gene name	Annotation
K01775	Alanine racemase *alr*	L-Alanine <=> D-Alanine
K01921	D-alanine-D-alanine ligase *ddl*	ADP-forming, ATP+2D-Alanine<=>ADP+Orthophosphate+D-Alanyl-D-alanine(ADP-forming)
K25316	Amino-acid racemase *racX*	L-lysine<=>D-lysine, L-Glutamine<=>D-Glutamine, L-Serine<=>D-Serine, L-Cysteine<=>D-Cysteine, L-Arginine<=>D-Arginine, L-Ornithine<=>D-Ornithine.
K01425	Glutaminase *gls*A, *gls*	D-Glutamine+H_2O<=>D-Glutamate+Ammonia
K01776	Glutamate racemase *murI*	L-Glutamate <=> D-Glutamate
K01925	D-glutamate ligase *murD*	UDP-N-acetylmuramoyl-L-alanine:D-glutamate ligase (ADP-forming)
K21898	Ornithine racemase *orr*	L-Ornithine <=> D-Ornithine
GE2163	Aspartate racemase *rac*D	L-Aspartate <=> D-Aspartate

Table S4. Primers used in the expression plasmid construction and saturation mutagenesis of RacX. The underlined sequences are *BamH*I and *Xho*I recognition sites, respectively.

Primer	Gene name	Description
racX-F	GGATCCATGAAAACTCTCGGAATGATC	*racX* cloning
racX-R	CTCGAGGTGACCTAAAATAAAATCC	*racX* cloning
C193S-F	GAATCGTTCTGGGCAGCACCGAGCTGCC	Cys193→Ser193
C193S-R	CGGCAGCTCGGTGCTGCCCAGAAC	Cys193→Ser193
A79C-F	GCCATTGTGATTTGTTGCAACACCCCGCAC	Ala79→Cys79
A79C-R	CAGGTGCGGGGTGTTGCAACAAATCAC	Ala79→Cys79
N80A-F	GTGATTTGTGCCGCCACCCCGCAC	Asn80→Ala80
N80A-R	CAGGTGCGGGGTGGCGGCACAAATC	Asn80→Ala80
T81A-F	GATTTGTGCCAACGCCCCGCACCTG	Thr81→Ala81
T81A-R	CCAGGTGCGGGGCGTTGGCAC	Thr81→Ala81
N121A-F	GCATCCTCGGCGCCAAGCCCAC	Asn121→Ala121
N121A-R	CATTGTGGGCTTGGCGCCGAGG	Asn121→Ala121
T124A-F	CGGCAACAAGCCCGCAATGACCG	Thr124→Ala124
T124A-R	GTTGCCGGTCATTGCGGGCTTG	Thr124→Ala124

Table S5. Comparative analysis of enzymatic activity between RacX racemase and other racemase.

Enzymes Source	Substrates	K_m (mM)	k_{cat} (s^{-1})	k_{cat}/K_m ($s^{-1}mM^{-1}$)	Reference
Halocola ammonii RacX	L-Lys	1.6±0.5	241.8± 12.4	151.2	This study
Pseudomonas putida Proline racemase	L-Pro	15.0±4.2	7.7±0.0	0.5	[1]
	D-Pro	5.3±1.1	69.6±8.6	13.1	
Thermococcus litoralis Racemase OCC_10945	L-Met	6.7 ± 0.5	1.1 ± 0.0	0.16	[34]
	L-Leu	2.3 ± 0.2	0.62 ± 0.0	0.28	
Thermus thermophilus Glutamate racemase	L-Glu	5.15 ± 0.6	17.7±0.9	3.4	[35]
	D-Glu	0.23 ± 0.0	1.1± 0.1	4.8	

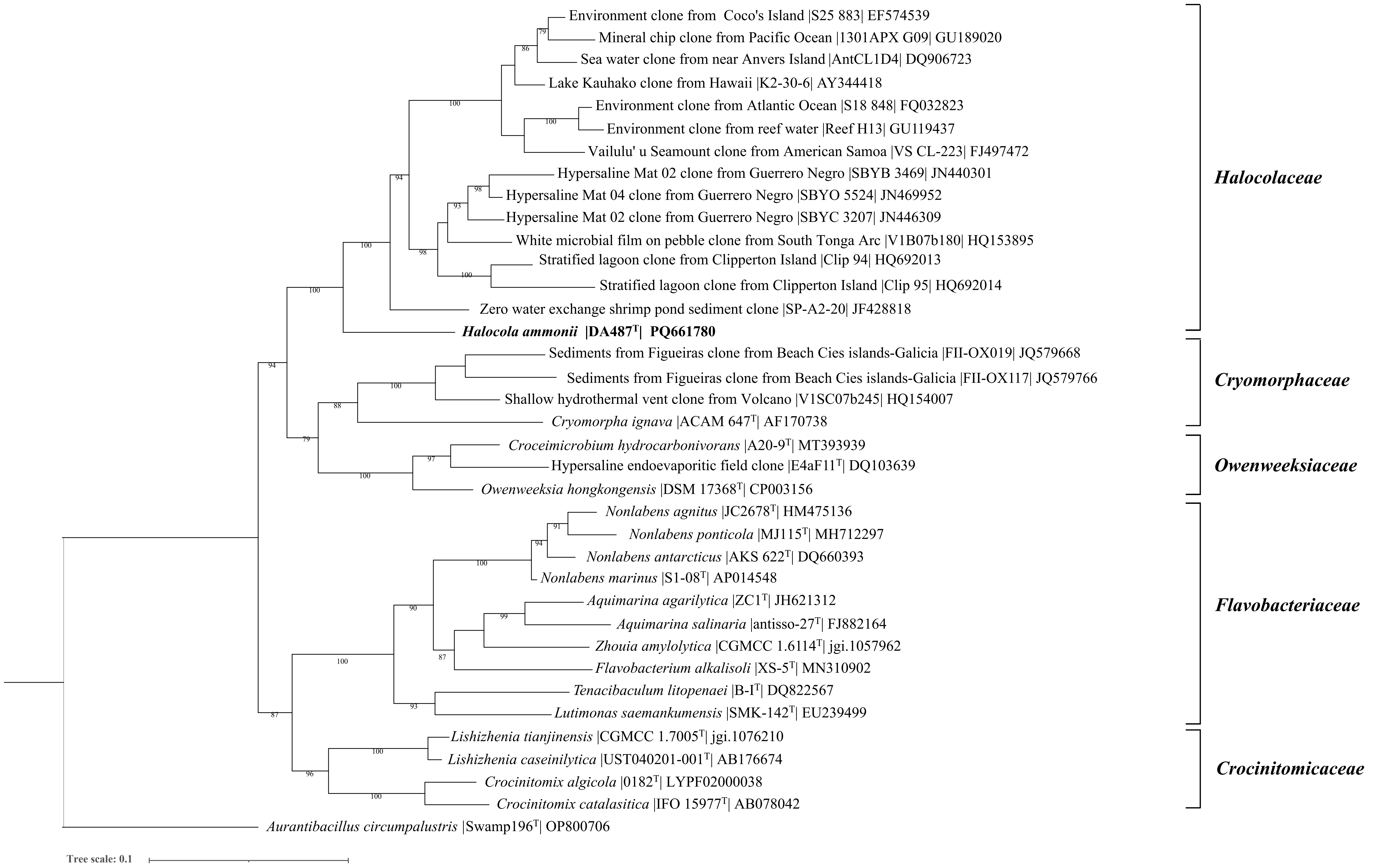

Fig S1 Maximum likelihood tree showing phylogenetic relationships based on the 16S rRNA gene sequences of DA487^T with closely related species of the order *Flavobacteriales*, including cultured representatives and environmental clones. *Aurantibacillus circumpalustris* Swamp196^T was used as the outgroup. Bootstrap values above 70% (1,000 replicates) are shown at branch nodes. Bar, 0.1 substitutions per nucleotide position.

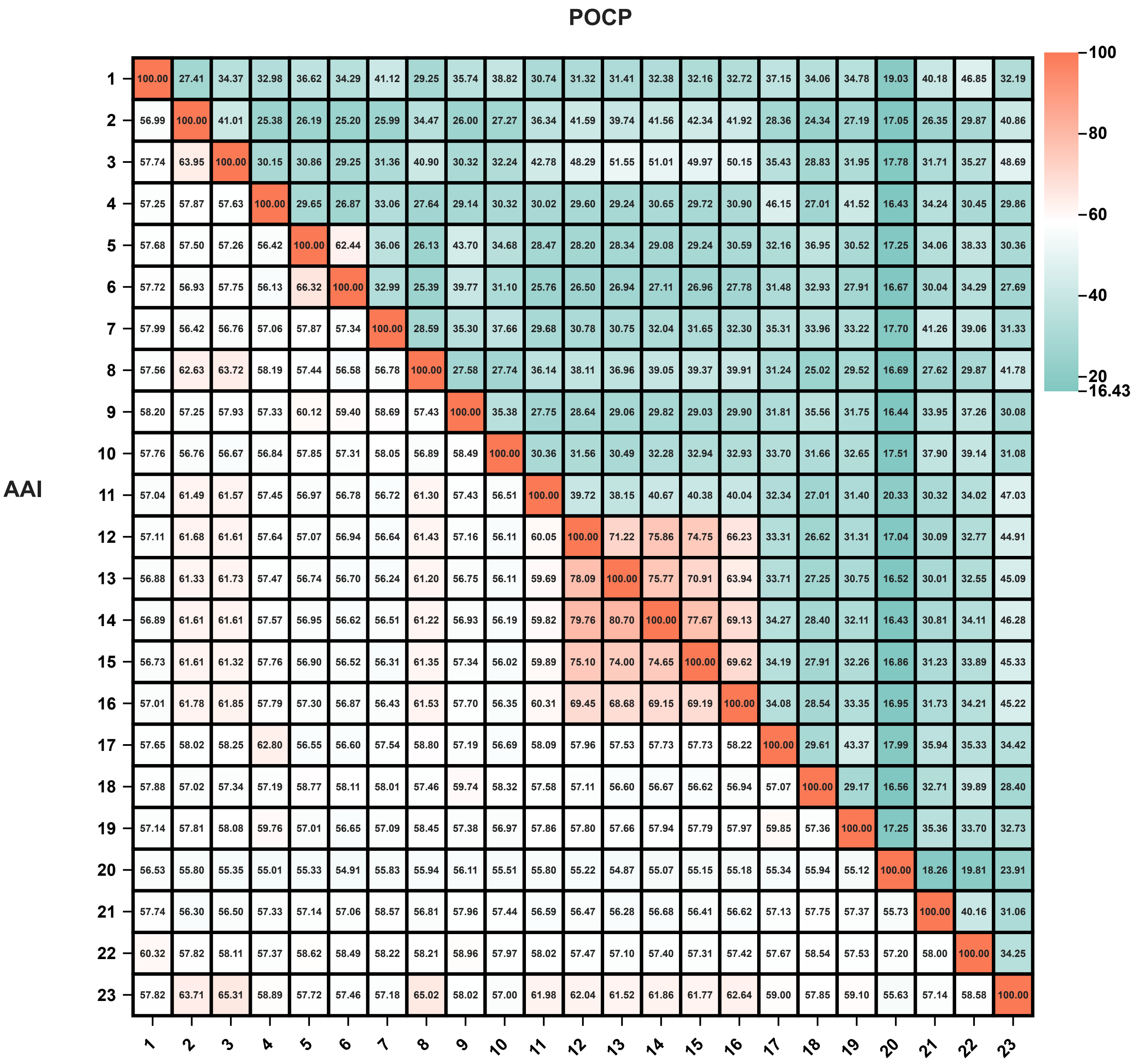

Fig S2 The analysis of the average amino acid identity (AAI) and the percentage of conserved proteins (POCP) values between *Halocola ammonii* DA487^{T} and cultured *Flavobacteriales* members ranged from 55.83%-58.69% and from 17.70%-41.26%, respectively. 1. *Acidiluteibacter ferrifornacis*, 2. *Aquimarina agarilytica*, 3. *Bizionia gelidisalsuginis*, 4. *Croceimicrobium hydrocarbonivorans*, 5. *Crocinitomix algicola*, 6. *Crocinitomix catalasitica*, 7. Strain DA487, 8. *Flavobacterium alkalisoli,* 9. *Lishizhenia tianjinensis*, 10. *Luteibaculum oceani*, 11. *Lutimonas saemankumensis*, 12. *Nonlabens agnitus*, 13. *Nonlabens antarcticus*, 14. *Nonlabens marinus,* 15. *Nonlabens ponticola*, 16. *Nonlabens tegetincola*, 17. *Owenweeksia hongkongensis,* 18. *Parvicella tangerina*, 19. *Phaeocystidibacter marisrubri*, 20. *Puteibacter caeruleilacunae*, 21. *Salibacter halophilus*, 22. *Vicingus serpentipes*, 23. *Zhouia amylolytica*

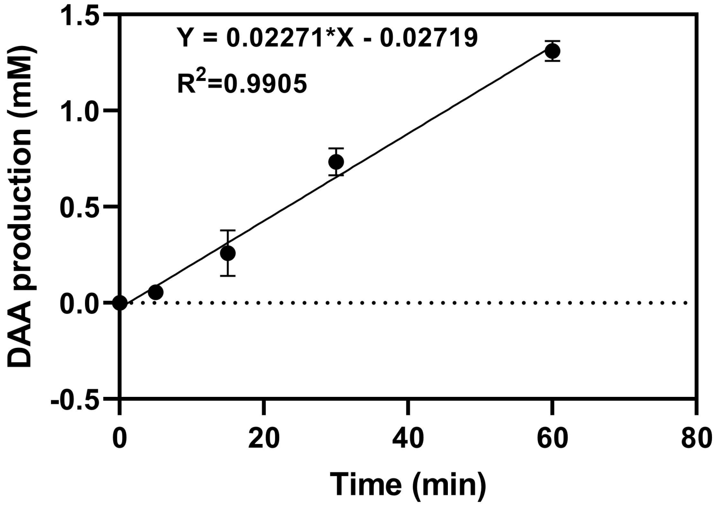

Fig S3 Time-course of D-Lys production by RacX. Reactions contained 5 mM L-Lys and 2.5 μg purified RacX in 50 mM Tris-HCl (pH 7.5) at 37°C. Data points represent mean ± SD (n=3). The solid line represents linear regression (0-30 min) with the equation Y = 0.0227* X - 0.02719 (R^2 = 0.9905).

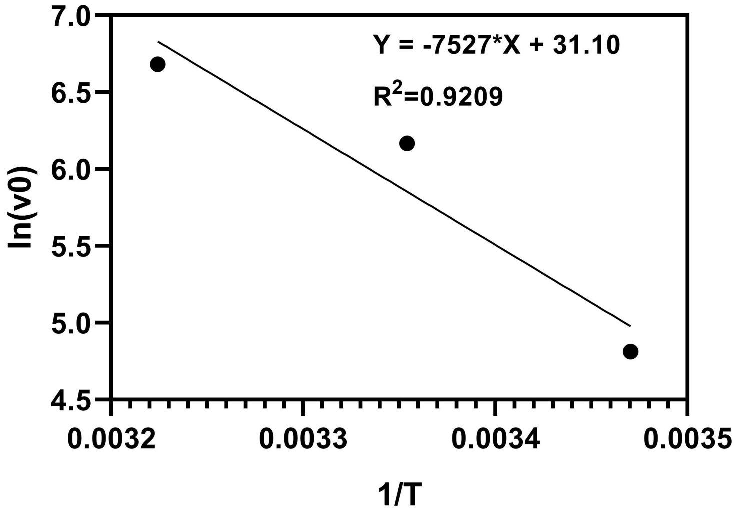

Fig S4 Arrhenius plot of RacX activity. The natural logarithm of the initial velocity ($\ln(v_0)$) is plotted against the inverse of absolute temperature (1/T) for the temperature range of 15°C to 37°C. The solid line represents the linear regression fit. The calculated activation energy (Ea) and the coefficient of determination (R^2) are displayed on the graph.

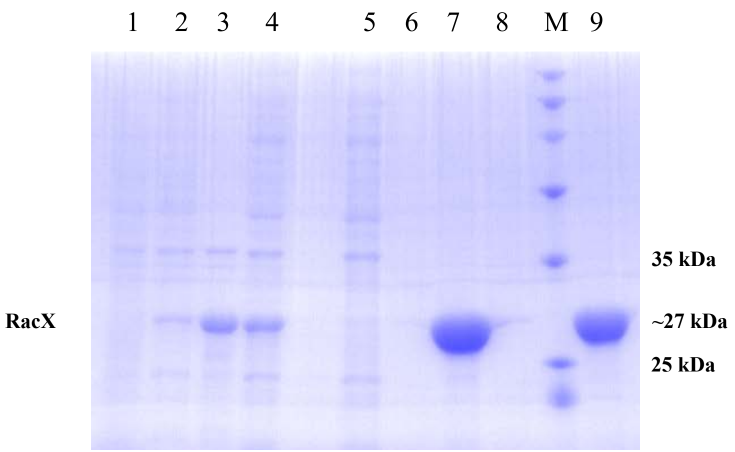

Fig S5 Detection of SDS-PAGE for the purification of RacX protein. 1. *E. coli Rosetta* strain, 2. Pellet after sonication, 3. Supernatant after sonication, 4. Flow-through of nickel column, 5. Lysis buffer containing 50 mM imidazole, 6. Elution buffer containing 80 mM imidazole, 7. Elution buffer containing 300 mM imidazole, 8. Elution buffer containing 500 mM imidazole, M. Marker, 9. Protein after dialysis.

RacX gene sequence

ATGAAAACTCTCGGAATGATCGGTGGCACCTCCTGGCACGCTACTATTGAGTACTATCG
ATTTATCAATGAAGGCGTGGCCAGGGAAATCGGCACGCAGGGAAATCCCCCACTCATC
ATCCACAGCATCAACATTGAGGTGATGCGATCTCAGGACAAGGACAGAATAGTTTCGA
CTTACACAGATGTGGCTAAAAAACTTGAAGCTGCCGGAGCTGAAGCCATTGTGATTTG
TGCCAACACCCCGCACCTGGTTTACAAAGAAGTGCAACCGCAGATCACAATTCCCTTT
TTGCACATCGCCAGAGCCACTGGCAAAGAGGCCCAAAAACTGGGACTCAAAAAGCTC
GGCATCCTCGGCAACAAGCCCACAATGACCGGCAACTTCATCCACAACATTATCGAAA
ATGAGTTTGAAATTGACCTCATCACTCCGCAGGGTGAGTCGCTAGATCGGTCGCACCA
CTTCGTTTCAAAAGAGTTGACACAGGGCGAATTCACCAAAGAGGCCCGCCAGTTTTAT
AAACGCGAAATTGAAAATCTGCAAAGCCGCGGTGCAGATGGAATCGTTCTGGGCTGC
ACCGAGCTGCCGATTTTATTGAAAGACCAGCAAAGTGAGATTCCGCTCCTTTCAACAA
CGCACCTGCACGCTCAAATGGCTGTGGATTTTATTTTAGGTCACTGA
